# Integrated Omic Analyses Provide Evidence that a “*Candidatus* Accumulibacter phosphatis” Strain Performs Denitrification under Microaerobic Conditions

**DOI:** 10.1128/mSystems.00193-18

**Published:** 2019-01-15

**Authors:** Pamela Y. Camejo, Ben O. Oyserman, Katherine D. McMahon, Daniel R. Noguera

**Affiliations:** a Department of Civil and Environmental Engineering, University of Wisconsin—Madison, Madison, Wisconsin, USA; b Department of Bacteriology, University of Wisconsin—Madison, Madison, Wisconsin, USA; University of California, San Diego

**Keywords:** Accumulibacter, RNA sequencing, denitrification, metagenomics, transcriptional regulation

## Abstract

The ability of “*Candidatus* Accumulibacter phosphatis” to grow and remove phosphorus from wastewater under cycling anaerobic and aerobic conditions has also been investigated as a metabolism that could lead to simultaneous removal of nitrogen and phosphorus by a single organism. However, although phosphorus removal under cyclic anaerobic and anoxic conditions has been demonstrated, clarifying the role of “*Ca*. Accumulibacter phosphatis” in this process has been challenging, since (i) experimental research describes contradictory findings, (ii) none of the published “*Ca*. Accumulibacter phosphatis” genomes show the existence of a complete respiratory pathway for denitrification, and (iii) some genomes lacking a complete respiratory pathway have genes for assimilatory nitrate reduction. In this study, we used an integrated omics analysis to elucidate the physiology of a “*Ca*. Accumulibacter phosphatis” strain enriched in a reactor operated under cyclic anaerobic and microaerobic conditions. The reactor’s performance suggested the ability of the enriched “*Ca*. Accumulibacter phosphatis” strain (clade IC) to simultaneously use oxygen and nitrate as electron acceptors under microaerobic conditions. A draft genome of this organism was assembled from metagenomic reads (“*Ca*. Accumulibacter phosphatis” UW-LDO-IC) and used as a reference to examine transcript abundance throughout one reactor cycle. The genome of UW-LDO-IC revealed the presence of a full pathway for respiratory denitrification. The observed transcript abundance patterns showed evidence of coregulation of the denitrifying genes along with a *cbb*_3_ cytochrome, which has been characterized as having high affinity for oxygen. Furthermore, we identified an FNR-like binding motif upstream of the coregulated genes, suggesting transcription-level regulation of both denitrifying and respiratory pathways in UW-LDO-IC. Taking the results together, the omics analysis provides strong evidence that “*Ca*. Accumulibacter phosphatis” UW-LDO-IC uses oxygen and nitrate simultaneously as electron acceptors under microaerobic conditions.

**IMPORTANCE** “*Candidatus* Accumulibacter phosphatis” is widely found in full-scale wastewater treatment plants, where it has been identified as the key organism for biological removal of phosphorus. Since aeration can account for 50% of the energy use during wastewater treatment, microaerobic conditions for wastewater treatment have emerged as a cost-effective alternative to conventional biological nutrient removal processes. Our report provides strong genomics-based evidence not only that “*Ca*. Accumulibacter phosphatis” is the main organism contributing to phosphorus removal under microaerobic conditions but also that this organism simultaneously respires nitrate and oxygen in this environment, consequently removing nitrogen and phosphorus from the wastewater. Such activity could be harnessed in innovative designs for cost-effective and energy-efficient optimization of wastewater treatment systems.

## Abstract (precis)

“*Candidatus* Accumulibacter phosphatis” is widely found in full-scale wastewater treatment plants, where it has been identified as the key organism for biological removal of phosphorus. Since aeration can account for 50% of the energy use during wastewater treatment, microaerobic conditions for wastewater treatment have emerged as a cost-effective alternative to conventional biological nutrient removal processes. Our report provides strong genomics-based evidence not only that “*Ca*. Accumulibacter phosphatis” is the main organism contributing to phosphorus removal under microaerobic conditions but also that this organism simultaneously respires nitrate and oxygen in this environment, consequently removing nitrogen and phosphorus from the wastewater. Such activity could be harnessed in innovative designs for cost-effective and energy-efficient optimization of wastewater treatment systems.

## INTRODUCTION

“*Candidatus* Accumulibacter phosphatis” (here referred to as “*Ca*. Accumulibacter phosphatis”) is the main microorganism removing phosphorus (P) in many wastewater treatment plants performing enhanced biological phosphorus removal (EBPR) (1–4). This uncultured polyphosphate-accumulating organism (PAO) fosters a unique and complex metabolism that responds to changes in the availability of carbon, phosphorus, and oxygen. Under anaerobic conditions, “*Ca*. Accumulibacter phosphatis” takes up volatile fatty acids (VFA) present in the wastewater and stores the carbon from these simple molecules intracellularly as poly-β-hydroxyalkanoate (PHA) while hydrolyzing intracellular polyphosphate to phosphate, which is then released from the cell to the liquid phase (5). The subsequent oxygen addition into the bulk liquid triggers the use of stored PHA molecules to generate energy for growth concomitant with phosphate uptake from the medium to form polyphosphate, eventually leading to the efficient removal of P from the wastewater.

Analysis of the “*Ca*. Accumulibacter phosphatis” lineage has led to the discovery of multiple genome variants. Using the polyphosphate kinase (*ppk1*) gene as a phylogenetic marker, “*Ca*. Accumulibacter phosphatis” variants have been subdivided into two types (types I and II) and 14 different clades (clades IA to E and IIA to I) (6–9). This genomic divergence may be responsible for the phenotypic variations of EBPR observed under different environmental conditions (10–14). Among these differences, the fitness of “*Ca*. Accumulibacter phosphatis” for anoxic respiration is a topic of much debate since published studies have presented contradictory findings on whether “*Ca*. Accumulibacter phosphatis” can respire nitrogenous compounds. In the case of “*Ca*. Accumulibacter phosphatis” type II, there is a general consensus that clade IIA is only capable of using nitrite as electron acceptor (10, 15–17). However, while several studies predicted that strains belonging to “*Ca*. Accumulibacter phosphatis” type I could use nitrite and/or nitrate as an electron acceptor (10, 15, 16, 18), other studies concluded that type I is not capable of anoxic nitrate respiration (19, 20). Those studies used different methods for clade classification, with some of them describing “*Ca*. Accumulibacter phosphatis” at the type level and others describing it at the clade level, as defined based on *ppk1* phylogeny (6). Therefore, it remains uncertain whether individual clades exhibit a consensus phenotype regarding respiration of nitrogenous compounds. It is also possible that the metabolic potential of “*Ca*. Accumulibacter phosphatis” may differ among strains within the same clade. Uncovering the metabolic traits characterizing distinct “*Ca*. Accumulibacter phosphatis” populations will provide a better understanding of the ecological roles played by distinct clades/populations and the biotechnological potential of this lineage in novel nutrient removal processes.

In a previous study, we characterized the “*Ca*. Accumulibacter phosphatis” clade-level population in a biological nutrient removal (BNR) reactor operated under cyclic anaerobic and microaerobic conditions and evaluated the ability of the enriched population to use multiple electron acceptors (8). Experimental evidence from this study led to the hypothesis that a particular “*Ca*. Accumulibacter phosphatis” clade (clade IC) could use oxygen and nitrate as electron acceptors (8) when the system is operated with cyclic anaerobic and microaerobic conditions. In this study, we used a combination of omics techniques to further investigate the genomic potential, gene expression, and transcriptional regulation of an enriched clade IC “*Ca*. Accumulibacter phosphatis” population to further elucidate the metabolic capabilities of this species-like group. The analysis provided strong evidence that the enriched “*Ca*. Accumulibacter phosphatis” clade IC population uses oxygen and nitrate simultaneously as electron acceptors under microaerobic conditions and that this organism therefore contributes to the simultaneous removal of nitrogen and phosphorus from wastewater.

## RESULTS AND DISCUSSION

### Characterization of reactor operation and “*Ca*. Accumulibacter phosphatis” community structure.

**FIG 1:**
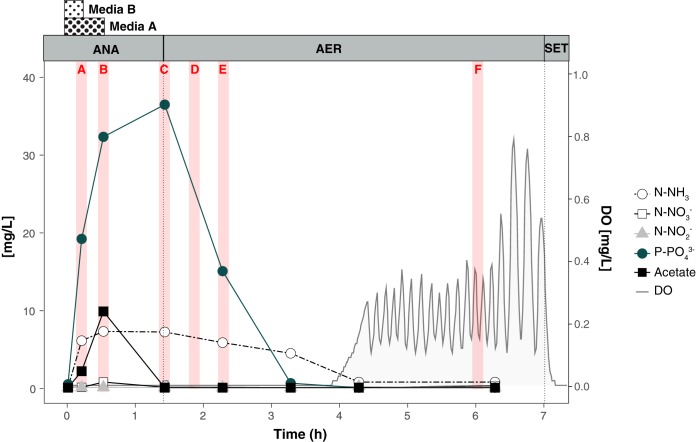
Nutrient profile of phosphorus, acetate, nitrogenous compounds, and oxygen concentration in the laboratory-scale SBR on day 522. Dotted lines separate the anaerobic (ANA), microaerobic (AER), and settling (SET) periods. Red bars indicate the time points used for RNA-seq (letters in red correspond to sample names). The periods of addition of media A (containing acetate and phosphate) and media B (containing ammonia) are indicated for the anaerobic stage.

We collected nutrient profiles across one reactor’s cycle on the same date that samples were collected for transcriptomics (day 522) (Fig. 1). Acetate was slowly added to the reactor during the first 32 min of the anaerobic phase; it was rapidly consumed, with only a small accumulation in the medium. P release to the mixed-liquor was observed during the acetate uptake period. During the anaerobic stage, ammonia-containing medium was supplied during the first 16 min of the anaerobic stage and the ammonia accumulated in the reactor, reaching a concentration of 8.5 mg N-NH_3_/liter (Fig. 1), which was in excess of what was required for heterotrophic growth, as discussed previously elsewhere (8). In the microaerobic phase, when the measured dissolved oxygen (DO) levels were about 0.02 mg/liter, P was taken up by cells. Simultaneously, nitrification occurred during the first 3 h of aeration, without NO_2_^-^ or NO_3_^-^ accumulation, indicating simultaneous nitrification and denitrification in the reactor. After all the substrates that imposed an oxygen demand were depleted, the level of oxygen increased and fluctuated around the 0.2 mg/liter set point. These observations are consistent with an efficient EBPR process under cyclic anaerobic and microaerobic conditions, as discussed elsewhere (8).

Samples collected on the same day for 16S rRNA gene amplicon sequencing indicated that “*Ca*. Accumulibacter phosphatis” was the most abundant bacterium in the reactor, accounting for 34% of the total number of reads (see Fig. S2 in the supplemental material). Members of the *Competibacteraceae* family (16% of the reads) and the *Lewinella* genus (11%) were also abundant. The diversity within the “*Ca*. Accumulibacter phosphatis” lineage was assessed by quantitative PCR (qPCR) (Fig. S2). In those samples, the “*Ca*. Accumulibacter phosphatis” members were dominated by clade IC, which accounted for 74% of the total, followed by clade IID (14%) and IIA (9%). As described before (8), clade IC predominated in the reactor during at least 300 days of operation, with abundances of more than 87% of the total “*Ca*. Accumulibacter phosphatis” level, and batch tests suggested its ability to use oxygen, nitrite, and nitrate as electron acceptors (8). Clade IC has also been described previously as the dominant “*Ca*. Accumulibacter phosphatis” clade in a reactor operated under anaerobic/anoxic/oxic conditions (19). However, in contrast to our findings, batch tests suggested that this strain was not capable of using nitrate as an external electron acceptor for anoxic P removal. This inconsistency among denitrifying capabilities could have been the result of genetic variations that are not captured with the current *ppk1*-based clade definitions.

### Assembling a draft genome of “*Ca*. Accumulibacter phosphatis” clade IC.

All existing metagenome-assembled genomes (MAGs) of “*Ca*. Accumulibacter phosphatis” have been obtained from bioreactors operated under conditions of conventional anaerobic/aerobic cycles that use abundant aeration (21–24). To date, only one genome of “*Ca*. Accumulibacter phosphatis” (the genome of clade IIA strain UW-1) has been closed, while draft genomes from 5 different clades (clades IA, IB, IIA, IIC, and IIF) have been reconstructed from metagenomic data. Since the sequencing batch reactor (SBR) operated with anaerobic/microaerobic cycles enriched for a less common clade of “*Ca*. Accumulibacter phosphatis,” we performed a metagenomic assessment of the microbial community in the reactor. The whole-community DNA from two reactor samples was sequenced using two different technologies: Illumina and Oxford Nanopore. Short Illumina reads were initially assembled and binned into 136 different bacterial draft genomes (see Table S1 in the supplemental material). One of these bins was classified as “*Ca*. Accumulibacter phosphatis” (bin.046) and characterized by its high completeness (94.8%) and relatively high redundancy (28.9%), likely due to the presence of redundant gene markers from other “*Ca*. Accumulibacter phosphatis” strains. The presence of another incomplete bin also classified as “*Ca*. Accumulibacter phosphatis” (bin.097.4; 26.0% completeness) further supports the idea that other “*Ca*. Accumulibacter phosphatis” strains were present at lower concentrations, in agreement with the diversity assessment based on the *ppk1* gene (Fig. S2). To obtain a higher-quality draft genome of the dominant “*Ca*. Accumulibacter phosphatis” strain, the differential coverage of two metagenomic samples was used to remove contaminant contigs, reducing the redundancy level to 0.84% of marker genes. Finally, Nanopore sequencing data were used for further scaffolding, helping reduce the number of scaffolds to half of the initial number, with a concurrent improvement in average contig length (Table S2). Gaps were filled using the GapCloser tool. The resulting near-complete draft genome, termed “*Ca*. Accumulibacter phosphatis” sp. UW-LDO-IC, has 4.7 Mbp in total with average GC content of 62.5% (Table S2) and harbored 95.2% of marker genes with 0.68% redundancy.

10.1128/mSystems.00193-18.6 TABLE S1 Bins assembled from metagenomic sample from day 522, taxonomic classification based on PhyloSift and metrics from CHECKM. Download Table S1, DOCX file, 0.1 MB. Copyright © 2019 Camejo et al. 2019 Camejo et al. This content is distributed under the terms of the Creative Commons Attribution 4.0 International license.

10.1128/mSystems.00193-18.7 TABLE S2 Metrics of “*Ca*. Accumulibacter phosphatis” UW-LDO-01 after each genome refinement step. Download Table S2, DOCX file, 0.01 MB. Copyright © 2019 Camejo et al. 2019 Camejo et al. This content is distributed under the terms of the Creative Commons Attribution 4.0 International license.

**FIG 2:**
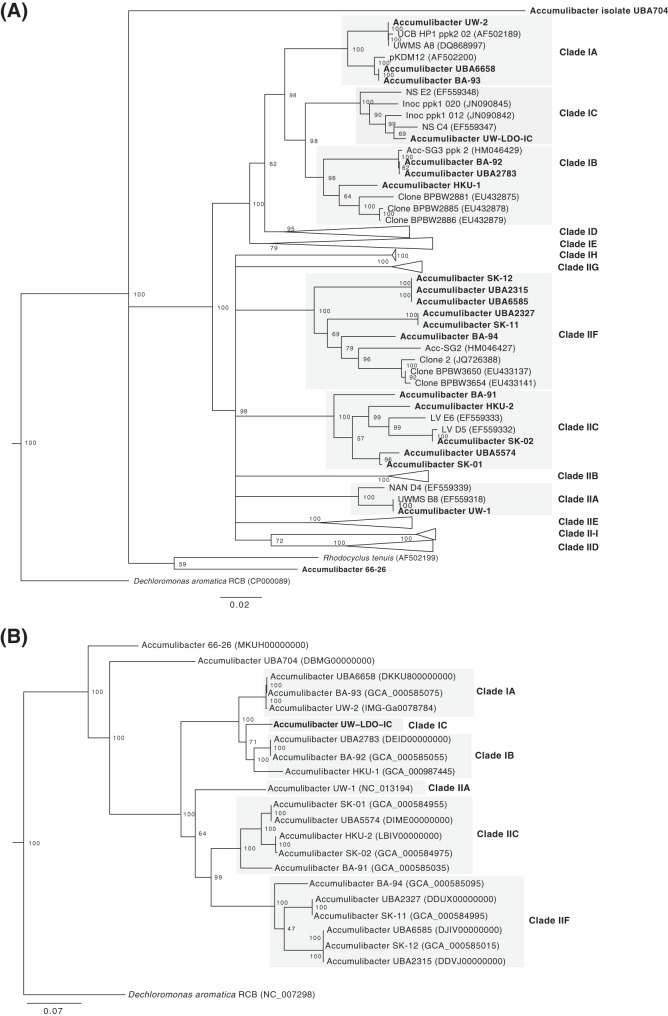
Phylogeny of “*Ca*. Accumulibacter phosphatis” UW-LDO-IC. (A) Neighbor-joining phylogenetic tree based on nucleic acid sequences of *ppk1* found in “*Ca*. Accumulibacter phosphatis” genomes. (B) RAxML phylogenetic tree of a concatenated alignment of 38 marker genes (nucleotide sequences) of the “*Ca*. Accumulibacter phosphatis” genus. Bootstrap values are shown in the tree branches based on 1,000 (A) and 100 (B) bootstrap replicates. The scale bars represent the number of nucleotide substitutions per site.

A phylogenetic tree constructed from the *ppk1* gene present in UW-LDO-IC and other “*Ca*. Accumulibacter phosphatis” genomes and sequences available at NCBI was used to classify UW-LDO-IC into 1 of the 14 “*Ca*. Accumulibacter phosphatis” clades described to date. According to the *ppk1* gene phylogenetic tree topology (Fig. 2A), *ppk1* of UW-LDO-IC clustered with sequences previously classified as clade IC; therefore, the draft genome assembled here would belong to this clade. With more “*Ca*. Accumulibacter phosphatis” draft genomes having become available in recent years, the tree topology also suggests that “*Ca*. Accumulibacter phosphatis” BA-92 (23), a draft genome initially classified as clade IC, might be better classified as belonging to clade IB along with the HKU-1 draft genome (22). To further evaluate this potential misclassification, a phylogenetic tree was constructed using a concatenated protein alignment of 38 universally distributed single-copy marker genes (25) (Fig. 2B). This tree topology supports the classification of UW-LDO-IC as type I but separate from “*Ca*. Accumulibacter phosphatis” BA-92 and HKU-1. Thus, we propose that UW-LDO-IC should be classified as the only draft genome representing clade IC and that “*Ca*. Accumulibacter phosphatis” BA-92 and HKU-1 should be classified in clade IB, along with “*Ca*. Accumulibacter phosphatis” UBA2783 (26).

Average nucleotide sequence identity (ANI) between UW-LDO-IC and the formerly published “*Ca*. Accumulibacter phosphatis” genomes was used to confirm the results of the phylogenetic analysis, as this method has been shown to correlate well with previously defined species boundaries (27, 28). The calculated ANI and alignment fraction for the “*Ca*. Accumulibacter phosphatis” genomes showed that UW-LDO-IC shares only 88.7% and 88.3% identity and 67.2% and 60.2% alignment with “*Ca*. Accumulibacter phosphatis” HKU-1 (clade IB) and BA-92 (clade IB), respectively (Fig. S1). The low levels of ANI and alignment, as well as the phylogeny corresponding to the concatenated markers, indicate that “*Ca*. Accumulibacter phosphatis” UW-LDO-IC shows significant differences from other “*Ca*. Accumulibacter phosphatis” genomes, none of which have been retrieved from BNR microbiomes adapted to minimal aeration.

10.1128/mSystems.00193-18.1 FIG S1 Comparison of the genome-wide average nucleotide identities and percentages of alignment of “*Ca*. Accumulibacter phosphatis”-like genomes. The heat map shows the average nucleotide identity (red upper section of matrix) and the minimum percentage of the two genomes that aligned (blue lower section). Download FIG S1, EPS file, 1.3 MB. Copyright © 2019 Camejo et al. 2019 Camejo et al. This content is distributed under the terms of the Creative Commons Attribution 4.0 International license.

10.1128/mSystems.00193-18.2 FIG S2 (Left) Estimated abundance, at the genus level, of the most abundant taxonomic units in sample used for RNA-seq (day 522). Values were determined using 16S rRNA amplicon sequencing, as described in Materials and Methods. (Right) Estimated abundance of each clade relative to the total “*Ca*. Accumulibacter phosphatis” lineage. Values were determined using qPCR targeting the *ppk1* gene, as described in Materials and Methods. Download FIG S2, EPS file, 0.9 MB. Copyright © 2019 Camejo et al. 2019 Camejo et al. This content is distributed under the terms of the Creative Commons Attribution 4.0 International license.

### Denitrification potential of “Ca. Accumulibacter phosphatis” UW-LDO-IC.

**FIG 3:**
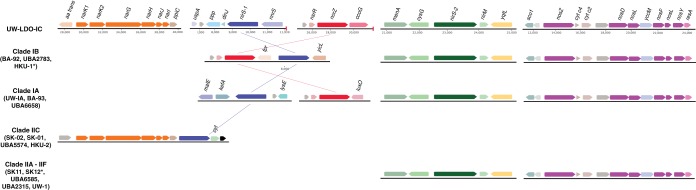
Schematic illustration of denitrifying gene loci in different genomes of “*Ca*. Accumulibacter phosphatis.” Changes in the positions of homologous genes are indicated by lines; the arrows show the direction of transcription. Genes are drawn to scale. Proteins with unknown function are colored gray and transposases black. The end of each scaffold is indicated with a red line. *aa trans*, amino acid carrier family; *nar*, nitrate reductase; *ppiC*, peptidyl-prolyl *cis*-*trans* isomerase C; *uspA*, universal stress protein A; *ppp*, protein phosphatase; *dinJ*, DNA-damage-inducible protein J; *nir*, nitrite reductase; *nsrR*, nitric oxide-sensitive repressor; *nor*, nitric oxide reductase; *ccoG*, type cbb3 cytochrome oxidase biogenesis protein; *menA*, 1,4-dihydroxy-2-naphthoate octaprenyltransferase; *cysG*, siroheme synthase; *yqfl*, ATP/GTP-binding protein; *sco1*, cytochrome *c* oxidase biogenesis protein; *nos*, nitrous oxide reductase; *cyt*, cytochrome; *resA*, cytochrome *c*-type biogenesis protein; *fpr*, ferredoxin-NADP reductase; *yicL*, carboxylate/amino acid/amine transporter; *malE*, carboxylate/amino acid/amine transporter; *kefA*, potassium efflux system protein; *lysE*, l-lysine exporter family protein; *luxO*, two-component signal response regulator. *, genome lacking one of the scaffolds.

A comparison of the genetic inventories involved in anoxic respiration revealed differences between the UW-LDO-IC and previously published “*Ca*. Accumulibacter phosphatis” genomes (Table 1; see also Table S5). Among the differences found is that UW-LDO-IC encodes a full respiratory denitrification pathway which involves a membrane-bound nitrate reductase operon of the NarG type (*narGHJI* operon), a nitrate/nitrite transporter gene homologous to the *narK* gene, three homolog genes encoding periplasmic cytochrome *cd_1_* nitrite reductases NirS (*nirS-1*, *nirS-2*, and *nirS-3*), and genes encoding the proteins involved in heme d_1_ biosynthesis (*nirMCFDLGHJN*), a quinol-dependent nitric oxide reductase *norZ* gene, and genes encoding a nitrous oxide reductase, Nos (*nosZDFYL*) (Table 1). The genetic context of these genes was compared to that of other “*Ca*. Accumulibacter phosphatis” genomes (Fig. 3). The positions of denitrifying genes within the genome differed among different clades. Other “*Ca*. Accumulibacter phosphatis” genomes contain one or two homologs of the *nirS* gene (Fig. S3B). Overall, genes flanking the *nar* operon, the *nirS-2* gene, and the *nosZ* gene were the same in all “*Ca*. Accumulibacter phosphatis” clades where these genes were identified, including UW-LDO-IC. Unlike the results seen with clade IIC and clade IB, *nirS-1* of UW-LDO-IC was not positioned next to the *nar* gene or *nor* gene, but the *nirS-1* genomic context in UW-LDO-IC differed from that in any “*Ca*. Accumulibacter phosphatis” genome.

**TABLE 1:**
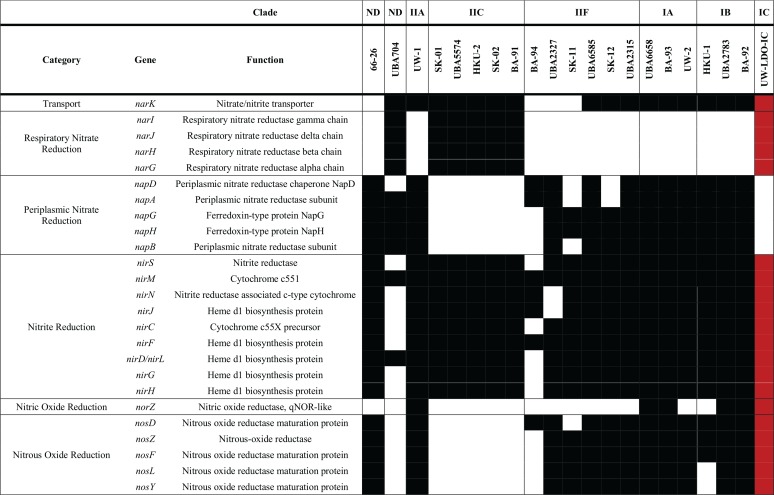
Inventory of genes associated with denitrification in complete and draft genomes of “*Ca*. Accumulibacter phosphatis”^*a*^

a Black and white rectangles represent the presence and absence of each gene, respectively. Genes present in the assembled “*Ca*. Accumulibacter phosphatis” sp. UW-LDO-IC genome are highlighted in red. ND, not determined.

10.1128/mSystems.00193-18.3 FIG S3 Neighbor-joining phylogenetic tree based on the full-length nucleic acid sequences of homologs to (A) *narG*, (B) *nirS*, (C) *norZ*, and (D) *nosZ* found in the genome of “*Ca*. Accumulibacter phosphatis” and annotated genomes in NCBI. Bootstrap values are shown in the tree branches based on 1,000 bootstrap replicates. The scale bar represents the number of nucleotide substitutions per site. Download FIG S3, EPS file, 2.7 MB. Copyright © 2019 Camejo et al. 2019 Camejo et al. This content is distributed under the terms of the Creative Commons Attribution 4.0 International license.

An alignment of full-length sequences of subunits *narG*, *nirS*, *norZ*, and *nosZ*, which contain the active sites corresponding to the encoded enzymes, was used to evaluate the phylogenetic associations of these genes (Fig. S3). This analysis revealed that all UW-LDO-IC genes involved in denitrification were closely related to the genes in other “*Ca*. Accumulibacter phosphatis” genomes, ruling out possible contig contamination from other denitrifying bacteria during binning. Phylogenetic differentiation between genes belonging to “*Ca*. Accumulibacter phosphatis” types I and II was observed, where genes belonging to UW-LDO-IC clustered with other genes from type I genomes. In the case of *narG*, which has been identified only in genomes from clade IIC, UW-LDO-IC clustered separately from the clade II genes (Fig. S3A).

Denitrifying genes present in “*Ca*. Accumulibacter phosphatis” genomes were phylogenetically related to different taxonomic groups. Genes encoding the NarG proteins seem to have derived from other *Betaproteobacteria*. Interestingly, these genes exhibit phylogenetic relationships with *narG* from different bacterial families (*Comamonadaceae*, *Pseudomonaceae*, *Burkholderaceae*, and *Rhodocyclaceae*), indicating similar origins (Fig. S3A). The close relationship of *narG* of “*Ca*. Accumulibacter phosphatis” with the plasmid-encoded gene in Burkholderia phymatum suggests potential mobility of this gene across genera.

Multiple *nirS* homologs are present in the majority of “*Ca*. Accumulibacter phosphatis” genomes, including UW-LDO-IC (Fig. S3B). The results of the phylogenetic analysis of sequences harboring this gene indicate that two main *nirS* clusters included sequences from both “*Ca*. Accumulibacter phosphatis” and other *Rhodocylaceae* family members, with one of these clusters, harboring the UW-LDO-IC *nirS-2* and *nirS-3* genes, being closely related to sequences from the *Pseudomonas* genus. Interestingly, only a few “*Ca*. Accumulibacter phosphatis” genomes contained a copy of a nitric oxide reductase *norZ* gene (Table 1), despite the fact that nitric oxide is assumed to be toxic to most organisms even at low concentrations (29). The lack of this gene in some strains might reflect either an incomplete assembly of those genomes or the importance of the flanking community in nitric oxide consumption. Furthermore, members of the *Rhodocyclaceae* family that are not related to the *Candidatus* “*Ca*. Accumulibacter phosphatis” genus do not encode NorZ. Instead, nitric oxide reduction in these other *Rhodocyclaceae* organisms requires the activity of a cytochrome *bc*-type complex (*norBC*). None of the “*Ca*. Accumulibacter phosphatis” genomes include the *norBC* genes, and the *norZ* genes of “*Ca*. Accumulibacter phosphatis” are phylogenetically most closely related to *Polaromonas*, a member of the *Comamonadaceae* family, from a different order within the *Betaproteobacteria* (Fig. S3C). The sequences closest to the *nosZ* gene of “*Ca*. Accumulibacter phosphatis” belonged to the *Dechloromonas*, in the *Rhodocyclaceae* family (Fig. S3D). These findings are in agreement with a recent ancestral genome reconstruction and evolutionary analysis of the “*Ca*. Accumulibacter phosphatis” lineage, which found that the denitrification machinery was not present in the last common ancestor of “*Ca*. Accumulibacter phosphatis” and that among the most abundant source of horizontally transferred genes are members of the *Burkholderiales* order within the *Betaproteobacteria*, including many from the *Comamonadaceae* family (see Table S6 in reference 30). Overall, these results suggest that part of the “*Ca*. Accumulibacter phosphatis” denitrification machinery was laterally transferred from other microorganisms commonly found in activated sludge.

Other “*Ca*. Accumulibacter phosphatis” genomes currently available do not have a complete respiratory denitrification pathway. Some have complete pathways for reducing nitrite to nitrogen gas (“*Ca*. Accumulibacter phosphatis” UW-1, UBA6658, UBA2783, UW-2, BA-92, and BA-93), and, instead of encoding the respiratory nitrate reductase (Nar), they encode the periplasmic nitrate reductase (Nap). Other genomes contain the Nar enzyme but lack the nitric oxide reductase (Nor) and therefore have the potential to reduce nitrate to nitric oxide (“*Ca*. Accumulibacter phosphatis” SK-01, SK-02, BA-91, UBA5574, and HKU-2) (Table 1). In contrast, “*Ca*. Accumulibacter phosphatis” UW-LDO-IC has a complete respiratory denitrification pathway but does not appear to have a periplasmic nitrate reductase (Table 1). Although Nar and Nap are both membrane-associated nitrate reductases, Nar is associated with the cytoplasm whereas Nap is associated with the periplasm (31). The location of Nar on the cytoplasmic side of the membrane predicts that nitrate respiration with Nar results in proton translocation and conservation of energy via ATP generation, which are essential requirements for nitrate respiration (31). In contrast, Nap’s location on the periplasmic side of the membrane predicts that nitrate reduction by this enzyme does not contribute to proton translocation or ATP generation. Therefore, the presence of Nap and absence of Nar in an organism cannot be assumed to result in nitrate respiration. Rather, nitrate reduction by Nap could lead to dissimilatory nitrate reduction to ammonia (31), could have a function on nitrate scavenging (32), or could help maintain redox balance in the cell (33).

Nevertheless, respiratory nitrate reduction via Nap, in the absence of Nar, has been demonstrated in *Epsilonproteobacteria* (34); therefore, it cannot be ruled out as a respiratory process in *Proteobacteria*, albeit with less ATP generation than with the Nar enzyme. In the specific case of “*Ca*. Accumulibacter phosphatis,” the presence of Nar and the rest of the respiratory pathway in UW-LDO-IC suggests that this organism is capable of respiratory nitrate reduction to nitrogen gas. This prediction helps settle the long-running debate in the research community about whether “*Ca*. Accumulibacter phosphatis” can achieve complete nitrate reduction to nitrogen gas while cycling polyphosphate (10, 15, 18, 35). Other “*Ca*. Accumulibacter phosphatis” species that have Nap and lack Nar have been hypothesized to also perform nitrate reduction to nitrogen gas (23), although it has also been shown that despite the existence of a *nap* operon within the “*Ca*. Accumulibacter phosphatis” UW-IA (clade IIA) genome (Table 1; see also Table S5), this strain cannot use nitrate as a terminal electron acceptor (10).

### Aerobic respiration potential of “*Ca*. Accumulibacter phosphatis” UW-LDO-IC.

The presence of known terminal oxidases, which catalyze oxygen reduction to water during the final step of the electron transport chain (36), was examined in the available “*Ca*. Accumulibacter phosphatis” genomes. Three known terminal oxidases were annotated in all “*Ca*. Accumulibacter phosphatis” genomes, namely, those of cytochrome *aa*_3_ (encoded by *ctaDCE*), *ba*_3_ (encoded by subunits *cbaA and cbaB*), and *cbb*_3_ (encoded by operon *ccoNOQP*) oxidases, which accept electrons from cytochrome *c* and transfer them in reactions involved in oxygen reduction (Table 2; see also Table S5). The *aa*_3_-type cytochrome *c* oxidases have low affinity for oxygen and usually play a dominant role under high-oxygen conditions (37–39). Phylogeny analysis of the enzyme’s first subunit (*ctaD*) (Fig. S4A) shows marked differentiation between the “*Ca*. Accumulibacter phosphatis” lineage and other *Rhodocyclaceae* organisms, although some “*Ca*. Accumulibacter phosphatis” genomes, including that of UW-I (clade IIA), clustered closely to *Dechloromonas* genomes. All genomes classified as clade IIF lacked this subunit (Table 2); thus, group members might rely on other cytochrome *c* oxidases for aerobic respiration.

**TABLE 2:**
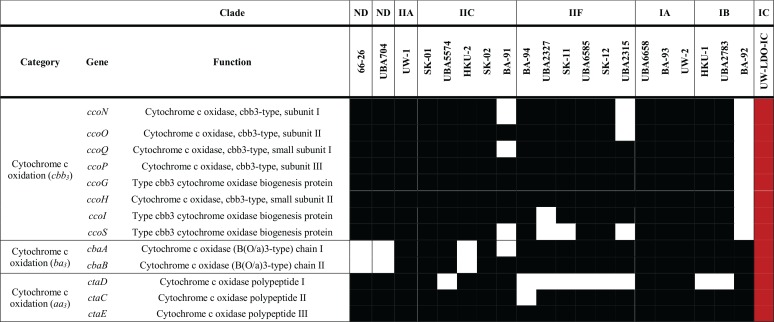
Inventory of genes associated with aerobic respiration in complete and draft genomes of “*Ca*. Accumulibacter phosphatis”^*a*^

a Black and white rectangles represent presence and absence of each gene, respectively. Genes present in the assembled “*Ca*. Accumulibacter phosphatis” sp. UW-LDO-IC genome are highlighted in red. ND, not determined.

10.1128/mSystems.00193-18.4 FIG S4 Neighbor-joining phylogenetic tree based on the full-length nucleic acid sequences of homologs to the cytochrome *c* oxidases (A) *aa*_3_ subunit I (*ctaD*), (B) c*bb*_3_ subunit I (*ccoN*), and (C) *ba*_3_ subunit I (*cbaA*) found in the genome of “*Ca*. Accumulibacter phosphatis” and annotated genomes in NCBI. Bootstrap values are shown in the tree branches based on 1,000 bootstrap replicates. The scale bar represents the number of nucleotide substitutions per site. Download FIG S4, EPS file, 2.6 MB. Copyright © 2019 Camejo et al. 2019 Camejo et al. This content is distributed under the terms of the Creative Commons Attribution 4.0 International license.

On the other hand, *cbb*_3_ oxidases are known to have very high affinity for oxygen and to be induced under low-oxygen conditions in many bacteria (40–44). This enzyme is widespread in the “*Ca*. Accumulibacter phosphatis” lineage, since 18 of the 21 genomes analyzed here harbored the *cco* operon encoding this enzyme’s subunits (Table 2). These results indicate that the ability to survive under conditions of low oxygen concentrations is common across “*Ca*. Accumulibacter phosphatis” strains; it could explain the high oxygen affinity (*k*_DO_) values reported for this organism (45, 46) and why lowering oxygen concentrations does not seem to negatively affect EBPR (8, 46, 47). The phylogenetic analysis of this enzyme’s first subunit, *ccoN*, shows conservation of this trait among *Rhodocyclaceae* family members (Fig. S4B).

Lastly, *ba*_3_*-*type cytochrome oxidase has been mostly studied in the extremophile bacterium Thermus thermophilus, where it is usually expressed under oxygen-limiting conditions (48), but little is known about its role(s) in other organisms. This enzyme is present in all clades in “*Ca*. Accumulibacter phosphatis” (Table 2), and its phylogeny reveals that it might have been derived from non-*Rhodocyclaceae* members (Fig. S4C).

### Changes in transcript abundance during an anaerobic/microaerobic cycle.

Transcriptional investigations in “*Ca*. Accumulibacter phosphatis” have illuminated how the complex and unique metabolism of this lineage is a result of highly dynamic gene expression (22, 49–51). Recently, the power of time series metatranscriptomics was used to analyze gene expression patterns in “*Ca*. Accumulibacter phosphatis” during an anaerobic-aerobic EBPR cycle (49). The study was carried out with high oxygen concentrations in a reactor where nitrification was inhibited and anoxic respiration did not take place. In order to study the effect of conditions of limited oxygen on the expression of the “*Ca*. Accumulibacter phosphatis” UW-LDO-IC respiratory machinery, we used a similar time series high-resolution transcriptome sequencing (RNA-seq) approach and contrasted the effects of high and low oxygen levels on the strain’s metabolism.

A time-series metatranscriptomic data set was obtained from the bioreactor. Samples were collected at the beginning of the anaerobic stage and at different times during the microaerobic stage when DO conditions were ∼0.05 mg/liter and 0.25 mg/liter, respectively (Fig. 1). RNA sequencing resulted in a total of 1,718,478,214 reads across the six samples (Table S3). Quality filtering, merging, and rRNA removal resulted in 396,995,401 sequences for downstream analysis. The resulting reads were then competitively mapped to “*Ca*. Accumulibacter phosphatis” UW-LDO-IC and other publicly available “*Ca*. Accumulibacter phosphatis” complete and draft genomes, including those corresponding to clades IA, IB, IIA, IIC, and IIF (Table S4). Between 48% and 50% of each sample’s filtered RNA reads mapped to the UW-LDO-IC genome, indicating that this was the most active bacterium in the community. No other “*Ca*. Accumulibacter phosphatis” genome retrieved more than 0.52% of mapping reads; therefore, strains closely related to other available “*Ca*. Accumulibacter phosphatis” genomes were not active community members.

10.1128/mSystems.00193-18.8 TABLE S3 RNA-seq summary. Data represent the number of reads after each step of RNA reads filtering and normalization factors used for RPKM calculations, as described in Materials and Methods. Download Table S3, DOCX file, 0.01 MB. Copyright © 2019 Camejo et al. 2019 Camejo et al. This content is distributed under the terms of the Creative Commons Attribution 4.0 International license.

10.1128/mSystems.00193-18.9 TABLE S4 Number and percentage of unambiguous RNA filtered reads mapping to the metagenomic assembly and “*Ca*. Accumulibacter phosphatis” genomes. Download Table S4, DOCX file, 0.02 MB. Copyright © 2019 Camejo et al. 2019 Camejo et al. This content is distributed under the terms of the Creative Commons Attribution 4.0 International license.

10.1128/mSystems.00193-18.10 TABLE S5 Locus tags of genes associated with denitrification and aerobic respiration in genomes of “*Ca*. Accumulibacter phosphatis” with published annotations. Download Table S5, DOCX file, 0.03 MB. Copyright © 2019 Camejo et al. 2019 Camejo et al. This content is distributed under the terms of the Creative Commons Attribution 4.0 International license.

**FIG 4:**
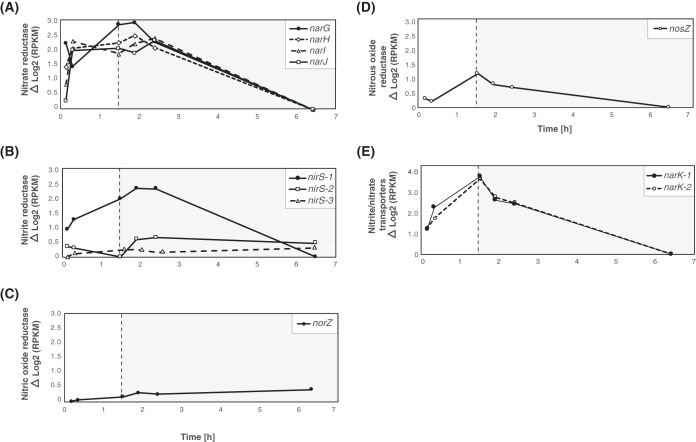
Gene expression profile patterns of denitrifying-related genes. Relative transcript abundances of (A) nitrate, (B) nitrite, (C) nitric oxide, and (D) nitrous oxide reductase genes and of (E) nitrite/nitrate transporter genes in “*Ca*. Accumulibacter phosphatis” UW-LDO-IC during the anaerobic (white panel) and microaerobic (gray panel) phases. The expression value corresponding to each time point was normalized to the minimum expression level of each gene over the cycle.

Transcripts mapping to genes related to denitrification were investigated by analyzing “*Ca*. Accumulibacter phosphatis” UW-LDO-IC gene expression patterns during the cycle. The data in Fig. 4 depict the relative expression levels of the genes encoding the *nar*, *nir*, *nor* and *nos* operon products and nitrite-nitrate transporters (*narK*) at each time point; the minimum expression level of each gene was subtracted from the level corresponding to each time point to allow better visualization of the dynamics of each transcript over the course of the cycle. All subunits of the *narGHJI* operon showed similar patterns, with transcript abundance increasing during the anaerobic stage, followed by a decrease in transcript levels as the oxygen concentration increased in the system at the end of the cycle (Fig. 4A). Only one of the three *nirS* homologs (*nirS*-1) present in “*Ca*. Accumulibacter phosphatis” UW-LDO-IC showed a pattern similar to that of the *narGHJI* operon (Fig. 4B), with an increase in transcript abundance during the anaerobic stage and a decrease of transcript abundance at the end of the aerobic stage. Levels of transcripts from the *narK* and *nosZ* genes also increased during the anaerobic stage, but their abundance started decreasing as soon as air was introduced into the reactor (Fig. 4D and E). The gene *norZ* did not display a notable change in relative transcript abundance over the course of the cycle [maximum Δlog_2_(RPKM) read count < 1] (where RPKM represents reads per kilobase per million), although its level of expression increased over time (Fig. 4C). These observations are consistent with the occurrence of denitrifying gene upregulation during the anaerobic stage, suggesting that the oxygen concentration plays an important role in transcriptional regulation of these genes, as has been previously described (52).

**FIG 5:**
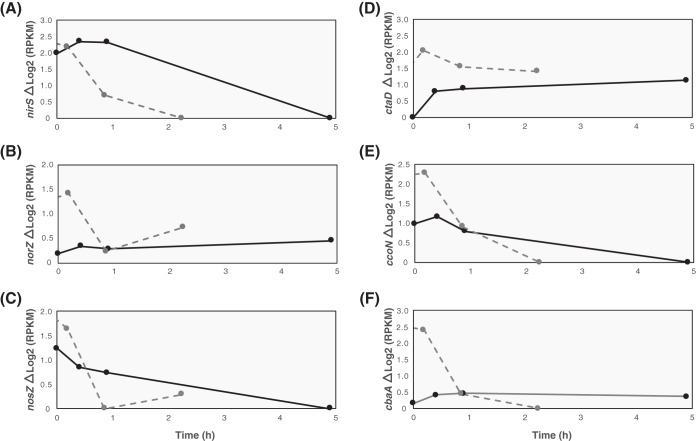
Gene expression comparison among the members of clades IC and IIA. Data represent the normalized transcript abundances of the (A) *nirS*, (B) *norZ*, and (C) *nosZ* denitrification-related genes and of the (D) *aa*_3_ cytochrome subunit I, (E) *cbb*_3_ subunit *ccoN*, and (F) *ba*_3_ subunit *cbaA* of “*Ca*. Accumulibacter phosphatis” UW-LDO-IC (solid line) and UW-1 (dotted line) (49) aerobic-respiration-related genes during the aerobic stage of an EBPR cycle.

Similar transcript trends for denitrification-associated genes have been previously described in “*Ca*. Accumulibacter phosphatis,” in reactors operating with anaerobic/aerobic cycles that used high aeration rates and where nitrification was chemically inhibited with allylthiourea (49, 50). In general, those studies showed denitrification gene upregulation under anaerobic conditions and a reduction in transcript abundance when oxygen was introduced to the system. However, unlike the results observed at high levels of DO (49), our observations indicate that transcript abundance remained high after oxygen addition, when nitrification and denitrification were occurring simultaneously. The *norZ* gene expression pattern seen under low-oxygen conditions also differed from the one reported at high DO levels (49), since the transcripts of this gene in UW-LDO-IC did not change considerably during the aerobic period, whereas the transcripts in UW-1 exhibited high variations during the operational cycle (Fig. 5B). Furthermore, the *nosZ* transcript levels seen under oxygen-limited conditions displayed a lower rate of decrease over the course of the cycle than had been reported to occur at high DO levels (49), where negligible expression was observed after 1 h of aeration (Fig. 5C); similar results of low expression at high DO levels were described previously (50). Since the complete denitrification pathway was not present in the “*Ca*. Accumulibacter phosphatis” UW-1 genome, no information about *nar* operon expression was available prior to our study.

Previous studies have confirmed the ability of “*Ca*. Accumulibacter phosphatis” to synthesize denitrification-associated proteins. In a previous report (21), nitrite and nitrous oxide reductase enzymes were detected by metaproteomic analysis of a “*Ca*. Accumulibacter phosphatis”-enriched microbial community. No nitric oxide reductase protein was detected in this study, despite the presence of the *norZ* gene in the genome of the strain enriched in this system, “*Ca*. Accumulibacter phosphatis” BA-93 (clade IA). As shown in Fig. 4C, the *norZ* transcript levels in UW-LDO-IC did not change considerably over time, potentially indicating a low transcription-level response to changes in oxygen or nitrite/nitrate concentrations; hence, other regulatory mechanisms might control synthesis of this enzyme. Further experiments are still needed to understand whether posttranscriptional regulation would also be controlling denitrification-associated protein synthesis in “*Ca*. Accumulibacter phosphatis.”

**FIG 6:**
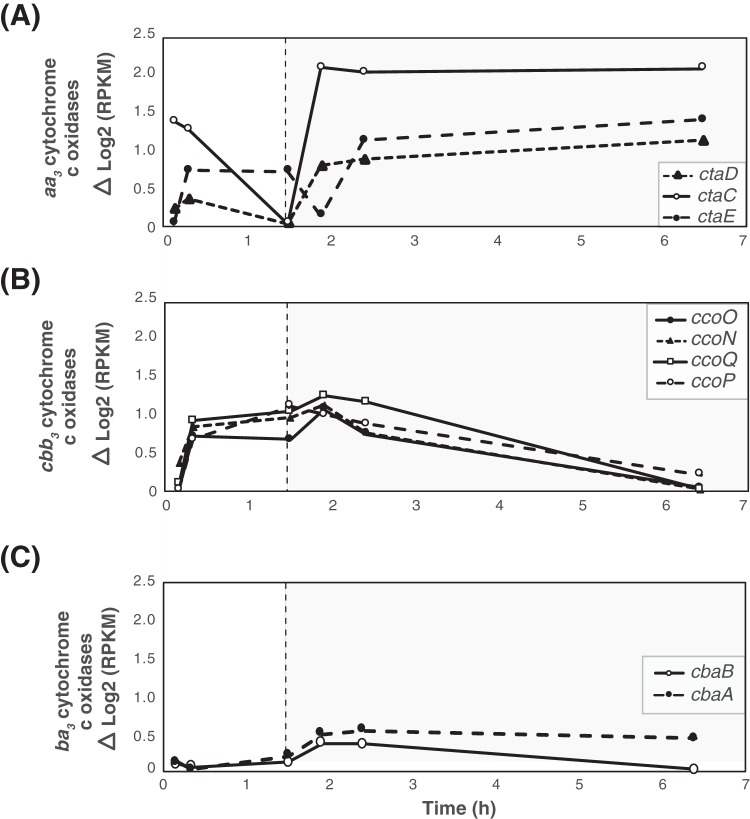
Gene expression profile patterns of aerobic-respiration-related genes. Data represent the relative levels of transcript abundance of (A) *aa*_3_, (B) *cbb*_3_, and (C) *ba*_3_ cytochrome *c* oxidases in “*Ca*. Accumulibacter phosphatis” UW-LDO-IC during the anaerobic (white panel) and microaerobic (gray panel) phases. The expression value corresponding to each time point was normalized to the minimum expression level of each gene over the cycle.

Changes in the expression levels of terminal oxidases in UW-LDO-IC were also identified. All subunits of the terminal cytochrome *c* oxidase *aa*_3_ subunits were upregulated during the entire microaerobic phase (Fig. 6A), indicating active aerobic respiration by this microorganism. Transcriptomic results also showed that the *cbb*_3_-type cytochrome oxidase transcripts decreased in level when DO levels increased in the reactor (Fig. 6B). Changes in cytochrome *ba*_3_ oxidase transcript levels were less pronounced than those observed in the other terminal oxidases [maximum Δlog_2_(RPKM) read count, <0.5] (Fig. 6C), likely indicating a minor role of this enzyme under conditions of redox variations.

We compared the levels of expression of these genes under conditions of high and low oxygen concentrations using data from a previous study (49) (Fig. 5). In both cases, the levels of low-affinity *aa*_3_ cytochrome oxidase transcriptional expression increased during the aerobic stage and remained upregulated until the cycle end (Fig. 5D). On the other hand, *cbb*_3_-type cytochrome oxidase transcript abundance drastically decreased after aeration was turned on in the high-aeration system, whereas the same gene in UW-LDO-IC remained upregulated during the first hour of minimal aeration, corroborating its function as a terminal oxidase induced under limited-oxygen conditions (Fig. 5E). Unlike UW-LDO-IC, “*Ca*. Accumulibacter phosphatis” UW-1 had high levels of *ba*_3_ cytochrome *c* oxidase expression during the anaerobic stages that declined after aeration started (Fig. 5F). Differences in the cytochrome expression profile of the latter suggest differential levels of gene expression control between these two clades.

Overall, these results demonstrated how expression patterns of the genes responsible for denitrification and aerobic respiration, specifically, *nar*, *nir*, and *cbb3*, showed upregulation during the beginning of the microaerobic stage, supporting our hypothesis that “*Ca*. Accumulibacter phosphatis” UW-LDO-IC is a denitrifying microorganism capable of simultaneously respiring oxygen and nitrate under microaerobic conditions.

### Validation of RNA-seq with RT-qPCR.

**FIG 7:**
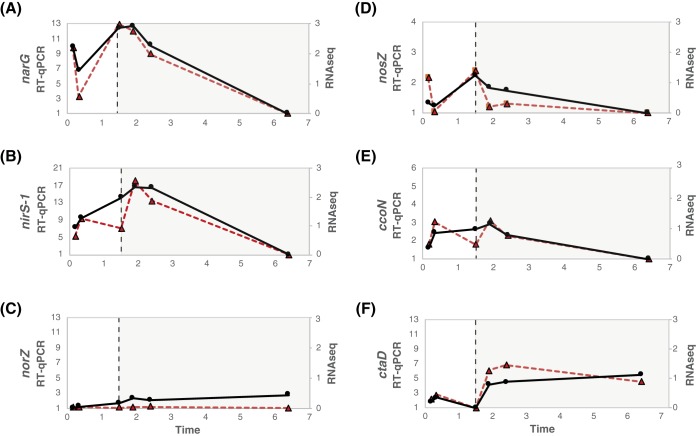
Comparison of the transcriptomic profiles obtained from RT-qPCR (gene copy numbers normalized by *rpoN* copy numbers; solid black lines) and RNA sequencing (Δlog_2_ RPKM; dotted red lines). Expression levels of genes involved in denitrification, i.e., *narG*, *nirS*, *norZ*, and *nosZ*, are presented in panels A, B, C, and D, respectively. Expression levels of genes involved in aerobic respiration, namely, *ccoN* (*cbb*_3_) and *ctaD* (*aa*_3_), are indicated in panels E and F, respectively.

Reverse transcription-quantitative PCR (RT-qPCR) was conducted using primers designed for six genes related to anoxic and aerobic respiration in UW-LDO-IC (Table 3), to validate the RNA sequencing results (Fig. 7). A no-reverse-transcription control (NRTC) was used to evaluate the background caused by trace DNA contamination. The average of the difference in *C_T_* (threshold cycle) values between the cDNA and NRTC was 10 cycles, indicating that DNA contamination was negligible. The copy number of the RNA polymerase sigma-54 factor, encoded by the *rpoN* gene, was used as a reference for normalization of the RT-qPCR data, since this gene showed no significant changes (ΔRPKM of <1) in the RNA sequencing results and has been previously used as a reference gene for qPCR normalization in other bacteria (53). The RT-qPCR transcriptomic profile in Fig. 6 was obtained by normalizing each point to the minimum number of copies across the cycle. In all cases, the gene regulation trends identified by RT-qPCR agreed with those determined by RNA sequencing (Fig. 7), corresponding to a Pearson correlation coefficient value of >0.5, except for *norZ*, where no significant changes in the expression level of this gene were quantified (fold change, <2).

**TABLE 3:** Summary of primers used for RT-qPCR

Target gene product	Gene symbol	Gene length (bp)	Primer target site	Primer sequence (5′–3′)	Amplicon length (bp)
Nitrite reductase	*nirS-1*	1,739	147−164	GTCGGCGATCGCGCAAT	164
			291−311	AGCGTTTTGTCCGTAGTCAGA	
Respiratory nitrate reductase alpha chain	*narG*	3,819	336−353	CCGATGGTTCGCGGTCA	91
			403−424	CGGACTCGACGATCGATGTCC	
Nitric oxide reductase, qNOR-like	*norZ*	2,282	1,846−1,864	CTTTGGGCTGGGTGGGT	375
			2,204−2,221	CGCGATCGGCGCTCAAAC	
Nitrous oxide reductase	*nosZ*	2,297	591−616	AAGAAATTCGAGCCACTAGAGGAAT	363
			935−954	AACATCCAGCGGCAATACG	
Cytochrome *c* oxidase, *cbb3*-type, subunit I	*ccoN*	1,428	241−260	CCTGCCATACGCGACTTTT	124
			344−365	TCGGCATACTCTTTGGACTGG	
Cytochrome *c* oxidase polypeptide I	*ctaD*	1,611	816−834	TCGCATGTCATCCCTGCC	135
			932−951	GGTCAGCGGAATACCGGTC	
RNA polymerase sigma-54 factor	*rpoN*	1,440	153−170	GAGTACGCGCGGACGGA	487
			594−611	TGTGCGCTGCGAGCCCC	

### FNR-type regulator controlling denitrification and aerobic respiration in “*Ca*. Accumulibacter phosphatis.”

**FIG 8:**
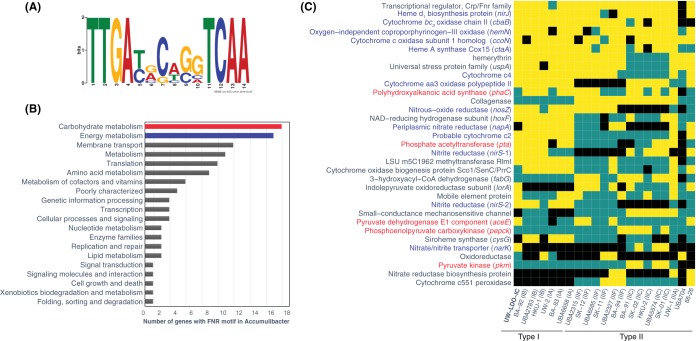
The FNR regulon in “*Ca*. Accumulibacter phosphatis.” (A) Motif diagram showing a putative FNR-binding site identified upstream of respiratory genes. (B) Distribution of KEGG functional categories in the fraction of genes with the FNR motif in genomes of “*Ca*. Accumulibacter phosphatis.” (C) The predicted conservation of the FNR regulon determined in the “*Ca*. Accumulibacter phosphatis” lineage; only genes with motifs present in at least 4 genomes are shown. Black indicates that the corresponding strain does not possess the corresponding gene; blue indicates that the strain possesses the gene but that an FNR motif was not found within the upstream intergenic region. Yellow indicates that the gene with an FNR motif is present in the genome.

Microbial respiration is usually regulated by an array of transcriptional factors, which respond to changes in the availability of electron acceptors in the environment to bind the genomic upstream region and modulate the expression of some of the components of the respiratory machinery. To identify putative transcriptional regulatory mechanisms of the respiratory machinery of “*Ca*. Accumulibacter phosphatis,” an upstream motif analysis was conducted. A sequence motif was identified in the intergenic regions upstream of the following genes with similar expression patterns: *nar*, *nir*, *nos*, and *cbb*_3_ operons of “*Ca*. Accumulibacter phosphatis” UW-LDO-IC (Fig. 8A). Comparison of this motif with the entries in the Prokaryote DNA motif database, using the scanning algorithm Tomtom (54) (*P* value, 3.04e−08), classified this sequence as the binding site of FNR, a relatively well-studied member of the CRP/FNR family of transcriptional regulators previously characterized in other proteobacteria (55). FNR is a global regulator of the anaerobic metabolism that has been reported previously to be necessary for denitrification (56) and aerobic respiratory pathway expression (57), and its activity is directly inhibited by oxygen via destruction of a labile iron-sulfur cluster (58). The presence of an oxygen-dependent transcriptional factor motif upstream of these operons would explain why upregulation of the genes comprising it was observed when no oxygen was present in the system (anaerobic stage), despite the lack of electron acceptors during this period.

Subsequent homology searches of this motif sequence were performed in the intergenic region of other “*Ca*. Accumulibacter phosphatis” genomes (*P* values, <1e−05). The results of the computational analysis predicted this motif to be located upstream of 165 different genes/operons across all genomes. According to the KEGG category III classification, the majority of these genes appear to be part of metabolic processes involved in carbohydrate and energy metabolism (Fig. 8B). Lists of genes with an FNR motif present in at least four “*Ca*. Accumulibacter phosphatis” genomes are presented in Fig. 8C. This analysis suggested that FNR participates in the regulation of many processes in “*Ca*. Accumulibacter phosphatis.” In addition to regulating the transcription of genes involved in denitrification and in the transcription of cytochrome *cbb*_3_ (as discussed above), FNR also regulates its own expression and regulates the expression of the *aa*_3_ and *ba*_3_ cytochrome oxidase operons required for aerobic respiration and for the biosynthesis of tetrapyrrole heme rings, which are prosthetic groups of many proteins involved in respiration and the metabolism and transport of oxygen (59) and in regulation of cytochromes *c*_2_ and *c*_4_, which are electron donors for *aa*_3_ and *cbb*_3_ oxidases (60, 61).

Interestingly, FNR also seems to participate in the regulation of carbon uptake, since in many cases, its binding site was positioned upstream of a phosphate acetyltransferase (*pta*) (Fig. 8C), an enzyme that participates in the conversion of acetate to acetyl-coenzyme A (acetyl-CoA) (62). This type of regulation has been observed before in Escherichia coli, where chromatin immunoprecipitation sequencing (ChIP-seq) tests revealed a putative binding site for FNR upstream of the *pta* operon (63) and RT-qPCR experiments in *fnr* mutants demonstrated that this regulator has a positive effect on the operon transcription (64). Although the FNR binding site was also located upstream of the PHA synthase gene (*phaC*) in multiple “*Ca*. Accumulibacter phosphatis” genomes (50), we did not observe changes in *phaC* transcript abundance after exposure to oxygen and, to our knowledge, FNR regulation has not been connected to PHA synthesis in other studied organisms. However, since regulation behaviors may differ among “*Ca*. Accumulibacter phosphatis” clades, further experiments still need to be carried out to evaluate the effect of oxygen on PHA accumulation in “*Ca*. Accumulibacter phosphatis.” Furthermore, in a few “*Ca*. Accumulibacter phosphatis” genomes, the FNR motif was also found upstream of genes encoding products involved in glycolysis/gluconeogenesis (pyruvate dehydrogenase E1 component [*aceE*], phosphoenolpyruvate carboxykinase [*pepck*], and pyruvate kinase [*pkm*]). Overall, these results provide evidence not only that oxygen-driven gene expression regulation is as an important factor in the adaptation of the metabolism of “*Ca*. Accumulibacter phosphatis” to low-DO and anoxic conditions but also that the oxygen concentration may directly influence the ability of “*Ca*. Accumulibacter phosphatis” to metabolize carbon.

Although oxygen gradients inside the flocs might influence the usage of different electron acceptors and potentially result in some “*Ca*. Accumulibacter phosphatis” cells using oxygen (in the outer parts of the floc) and others using nitrate (in the inner parts of the floc), the presence of FNR motifs upstream of denitrification genes and of the high-affinity *cbb*_3_ cytochrome used for oxygen respiration is in agreement with the hypothesis that “*Ca*. Accumulibacter phosphatis” is capable of using the two electron acceptors simultaneously under low-oxygen conditions. Moreover, the fact that simultaneous respiration of nitrate/nitrite and oxygen has been formerly observed in other microorganisms (65–68) further supports this hypothesis. Thus, even with anoxic microniches inside the flocs, the observations are consistent with the capability of the enriched “*Ca*. Accumulibacter phosphatis” to perform corespiration of oxygen and nitrate.

**FIG 9:**
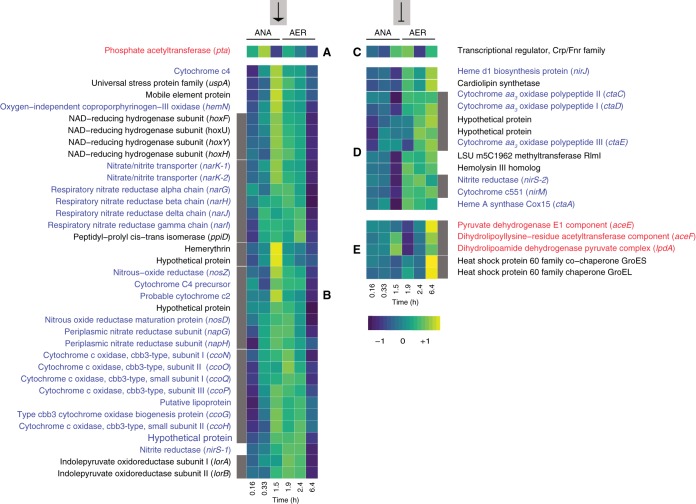
Transcription profile heat map of members of the FNR regulon in UW-LDO-IC. The colors represent the relative levels of mRNA abundance at each time point of the cycle compared to the mean level of expression (yellow, high expression; dark blue, low expression). Genes were clustered according to their expression profiles. Lateral gray bars indicate genes belonging to the same operon. Groups A and B contain genes whose expression levels negatively correlate with oxygen tension (positively regulated by FNR). Groups C, D, and E contain genes upregulated during periods of high oxygen levels (negatively regulated by FNR). ANA, anaerobic; AER, microaerobic.

The transcriptional patterns of genes with putative FNR binding sites in UW-LDO-IC were analyzed for oxygen-dependent changes in transcript abundance. The expression profiles of regulated genes and operons were grouped by similarity (Pearson correlation), resulting in 6 clusters with different patterns of transcript abundance over the course of the cycle (Fig. 9). Two of these clusters were associated with positive control by FNR. That is, they showed increases in transcript abundance during the early anaerobic (cluster A) or late anaerobic (cluster B) stages, when the FNR regulator is active. These clusters included genes associated with anaerobic respiration (*nar* and *nos* operons, *nirS-1*), microaerobic respiration (*cco* operon and *hemN*), electron transport (cytochrome *c*_2_ and *c*_4_), response to stress (*uspA*), oxygen detection (hemerythrin), and acetate uptake (*pta*). In contrast, clusters D and E were characterized by lower transcript abundance when the FNR regulator was active, meaning that they were negatively regulated (expression was repressed) by FNR and that their expression increased at high levels of oxygen, when the FNR regulator was inactive (Fig. 9). Several genes associated with aerobic respiration are included in cluster D, such as the genes encoding low-oxygen-affinity cytochrome *aa*_3_ and *ba*_3_ oxidases and a protein involved in the synthesis of heme A, a prosthetic group required by cytochrome *a*-type respiratory oxidases. A *nirS* homolog, *nirS-2*, also clustered within this group, displaying low transcript abundance under anaerobic conditions and higher transcript abundance under high-oxygen conditions (Fig. 9). This pattern of regulation suggests that *nirS-2* is not participating in anaerobic nitrate respiration in “*Ca*. Accumulibacter phosphatis” UW-LDO-IC, while *nirS-1*, which is in cluster B, may be the only active nitrite reductase in this organism. Since many “*Ca*. Accumulibacter phosphatis” genomes indicate the presence of at least two *nirS* homologs (Fig. S3), it may be possible that the nitrite reductase activity in those strains is also restricted to only one of the homologs. Cluster C has a unique transcriptional profile and contains only one gene, the transcriptional regulator FNR gene (Fig. 9). Its unique pattern might be attributed to the effect of autoregulation that FNR is known to have on its own transcription under anaerobic conditions (69).

The large number of expression patterns observed in genes putatively regulated by FNR suggests that the transcription of some of these genes could be affected by additional regulatory mechanisms, such as combinatorial binding of other regulators and/or epigenetic signals (70). Other transcriptional factors encoded by genes identified in the “*Ca*. Accumulibacter phosphatis” genomes, including NreABC, NsrR, NorR, and RegAB, might also sense low-oxygen conditions and regulate part of the anoxic and aerobic respiratory pathways, as has been described previously in other microorganisms (71–74). However, at this time, no binding sites associated with any of these additional regulatory elements have been identified in “*Ca*. Accumulibacter phosphatis.”

Overall, this report dissects the metabolic response of “*Ca*. Accumulibacter phosphatis” to oxygen-limited conditions. The comparative genomic results provide evidence for the unique respiratory machinery encoded in the newly assembled genome of “*Ca*. Accumulibacter phosphatis” UW-LDO-IC, which confers to this strain the capability to simultaneously reduce oxygen and nitrogenous compounds. Simultaneous upregulation of aerobic and anoxic respiratory pathways and coregulation by the FNR transcriptional factor further support the idea of the use of multiple electron acceptors by UW-LDO-IC. Further studies should include experiments analyzing the transcriptional regulation of these pathways at the genome-scale level, using modern approaches such as transcriptional regulatory networks (TRN) and genome-wide binding site location methods, such as chromatin immunoprecipitation (ChIP) sequencing (75) and DNA affinity purification (DAP) sequencing (76).

## MATERIALS AND METHODS

### Operation of laboratory-scale sequencing batch reactor.

A laboratory-scale SBR was used in this study. The reactor was originally inoculated with activated sludge obtained from the Nine Springs wastewater treatment plant in Madison, WI, which uses a variation of the University of Cape Town (UCT) process designed to achieve biological P removal without nitrate removal (77) from municipal wastewater and operates with high aeration rates (DO values ranging from 0.8 to 4.5 mg/liter) (47). Details of the laboratory-scale operation under cyclic anaerobic and microaerobic conditions were provided in a previous report (8). Briefly, the SBR had a 2-liter working volume and was fed with synthetic wastewater containing acetate (500 mg chemical oxygen demand [COD]/liter) as the sole carbon source (C/P molar ratio of 20). The synthetic wastewater was dispensed as two separate media; medium A contained the acetate and phosphate, whereas medium B supplied the ammonia (8). The reactor was operated under conditions of alternating anaerobic and low-oxygen 8-h cycles. Each cycle consisted of 1.5 h anaerobic stage, 5.5 h microaerobic stage, 50 min settling, and 10 min decanting. During the microaerobic stage, an on/off control system was used to limit the amount of oxygen pumped to the reactor (0.02 liters/min) and to maintain microaerobic conditions in the mixed liquor (DO set point = 0.2 mg/liter). The hydraulic retention time (HRT) and solid retention time (SRT) were 24 h and 80 days, respectively. The pH in the system was controlled to be between 7.0 and 7.5.

### Sample collection and analytical tests.

To monitor reactor performance, mixed liquor and effluent samples were collected, filtered through a membrane filter (Whatman, Maidstone, United Kingdom) (0.45-μm pore size), and analyzed for acetate, PO_4_^3-^-P, NH_3_ plus NH_4_^+^-N, NO_3_^-^-N, and NO_2_^-^-N. The concentrations of PO_4_^3-^–P were determined according to standard methods (78). Total ammonia (NH_3_ plus NH_4_^+^) concentrations were analyzed using the salicylate method (method 10031; Hach Company, Loveland, CO). Acetate, nitrite, and nitrate levels were measured using high-pressure liquid chromatography as previously described (8).

For 16S rRNA-based tag sequencing and metagenomic analyses, biomass samples from the reactors were collected weekly and stored at −80°C until DNA extraction was performed. DNA was extracted using an UltraClean soil DNA isolation kit (MoBIO Laboratories, Carlsbad, CA). Extracted DNA was quantified using a NanoDrop spectrophotometer (Thermo Fisher Scientific, Waltham, MA) and stored at −80°C.

For transcriptomic analyses, biomass samples were collected across a single reactor cycle to capture key transition points in the EBPR cycle (Fig. 1). Samples (2 ml) were collected in microcentrifuge tubes and centrifuged, the supernatant was removed, and the cell pellets were flash frozen in a dry ice and ethanol bath within 3 min of collection. RNA was extracted from the samples using an RNeasy kit (Qiagen, Valencia, CA, USA) with a DNase digestion step. RNA integrity and DNA contamination were assessed using an Agilent 2100 Bioanalyzer (Agilent Technologies, Palo Alto, CA, USA).

### rRNA gene-based tag sequencing.

The composition of the microbial community in the reactor was determined via the analysis of high-throughput sequencing of 16S rRNA gene fragments. The hypervariable V3-V4 regions of the bacterial 16S rRNA gene were amplified using primers 515f/806r (79) as described previously (8). The sequencing data are available under BioProject PRJNA482250. Briefly, PCR products were generated using an *Ex Taq* kit (TaKaRa); cycling conditions involved an initial 5-min denaturing step at 95°C, followed by 35 cycles of 95°C for 45 s, 50°C for 60 s, and 72°C for 90 s and a final elongation step at 72°C for 10 min. Amplicons were visualized on an agarose gel to confirm product sizes. Purified amplicons were pooled in equimolar quantities and sequenced on an Illumina Miseq benchtop sequencer using paired-end 250-bp kits at the Cincinnati Children’s Hospital DNA Core facility.

The paired-end reads that were obtained were merged, aligned, filtered, and binned into operational taxonomic units (OTU) with 97% identity using the QIIME pipeline (80). Chimeric sequences were removed using UCHIME (81). The most representative sequences from each OTU were taxonomically classified using the MIDas-DK database (82).

### Quantitative PCR (qPCR).

Quantification of each “*Ca*. Accumulibacter phosphatis” clade was carried out by qPCR using a set of clade-specific primers targeting the polyphosphate kinase (*ppk1*) gene (8). All qPCR reactions were run in a LightCycler 480 system (Roche Applied Science, Indianapolis, IN). The volume of each reaction mixture was 20 μl, and each reaction mixture contained 10 μl iQSYBR green Supermix (Bio-Rad Laboratories, Hercules, CA), 0.8 μl each of 10 μM forward and reverse primers, 4.4 μl of nuclease-free water, and 4 μl of sample. Templates for qPCR were obtained from clone collections or gene synthesis (IDT, USA). In all cases, 10-fold serial dilutions of each template (ranging from 10^1^ to 10^7^ copies per reaction) were used to generate qPCR calibration curves. All samples were processed in triplicate, and each reaction plate contained nontemplate controls.

### Metagenome sequencing, assembly, and binning.

Samples from days 522 and 784 were selected for metagenomic analysis. Illumina TruSeq DNA PCR-free libraries were prepared for DNA extracts according to the manufacturer’s protocol and subjected to paired-end sequencing on either an Illumina HiSeq 2000 platform (v4 chemistry, 2 by 150 bp; day 522 sample) or an Illumina MiSeq platform (v3 chemistry, 2 by 250 bp; day 784 sample). Sequencing of these samples generated 17 and 2.6 Gb of data for the day 522 and day 784 samples, respectively. A sample from day 522 was also sequenced using MinION (Oxford Nanopore Technologies, Oxford, United Kingdom), according to the Oxford Nanopore Genomic DNA Sequencing protocol (SQK-MAP003). The MinION flow cell was run for 48 h using MinION control software (MinKNOW [version 47.3]), and online base-calling was performed using Metrichor software (version 2.23) (229.2 megabases were generated). Raw reads have been submitted to NCBI and are accessible under the BioProject identifier PRJNA322674.

Illumina unmerged reads were quality-trimmed and filtered with Sickle (https://github.com/ucdavis-bioinformatics/sickle.git), using a minimum Phred score of 20 and a minimum length of 50 bp. Metagenomic reads from day 522 were assembled using the metaSPAdes pipeline of SPAdes 3.9.0 (83), and individual genome bins were extracted from the metagenome assembly using MaxBin (84). Genome completeness and redundancy were estimated using CHECKM 0.7.1 (85). The taxonomic identity of the bins was assigned using PhyloSift v 1.0.1 (86) and the script “parse_phylosift_sts.py” (available at https://github.com/sstevens2/sstevens_pubscrip/blob/master/parse_phylosift_sts.py) (with options -*co_prob* 0.7 and -*co_perc* 0.7). Bin information is summarized in Table S1 in the supplemental material.

Two putative “*Ca*. Accumulibacter phosphatis” bins (bin.046 and bin.097.4 in Table S1) were identified. The bin with the highest completeness (bin.046; here referred to as UW-LDO-IC) was selected for further analysis and subjected to further processing to improve its quality. Redundant scaffolds were manually removed based on tetranucleotide frequency and differential coverage, using metagenomic reads from days 522 and 784 and following the anvi’o workflow described previously by Eren et al. (87). An image of the phylogram generated with scaffolds of bin.046, using the function “anvi-interactive” (order of items, Seq. Composition + Diff Coverage; view, Main coverage), is shown in Fig. S5 in the supplemental material. This phylogram exhibits separation of bin.046 into two branches, with one of them corresponding to UW-LDO-IC. Further scaffolding was performed on UW-LDO-IC using Nanopore long reads and the LINKS algorithm (88). Gapcloser (https://sourceforge.net/projects/soapdenovo2/files/GapCloser/) was used for additional gap filling. Table S2 displays quality metrics of the “*Ca*. Accumulibacter phosphatis” draft genome after each of the steps previously described. The metagenomic assembly and final “*Ca*. Accumulibacter phosphatis” bin were annotated using MetaPathways v 2.0 (89) and can be found under GenBank accession number QPGA00000000.

10.1128/mSystems.00193-18.5 FIG S5 Circle phylogram generated with scaffolds of bin.046, using the function “anvi-interactive” (order of items, Seq. Composition + Diff Coverage; view, Main coverage). Two main branches are highlighted, showing in red the branch that corresponds to UW-LDO-IC. Download FIG S5, EPS file, 2.9 MB. Copyright © 2019 Camejo et al. 2019 Camejo et al. This content is distributed under the terms of the Creative Commons Attribution 4.0 International license.

### Average nucleotide identity (ANI).

Pair-wise ANI values of “*Ca*. Accumulibacter phosphatis” genomes were obtained using the ANIm method (90) and implemented in the Python script “calculate_ani.py” available at https://github.com/ctSkennerton/scriptShed/blob/master/calculate_ani.py.

### Phylogenetic analyses.

The phylogeny of “*Ca*. Accumulibacter phosphatis” UW-LDO-IC was assessed by constructing a phylogenetic tree using a concatenated alignment of marker genes. Published “*Ca*. Accumulibacter phosphatis” draft and complete genomes were included in the analysis. First, PhyloSift was used to extract a set of 38 marker genes from each genome. Then, the extracted marker protein sequences were concatenated into a continuous alignment to construct a maximum-likelihood (ML) tree, using RAxML v 7.2.8 (91). RAxML was used to generate 100 rapid bootstrap replicates followed by a search for the best-scoring ML tree.

For phylogenetic analyses of the polyphosphate kinase 1 (*ppk1*), nitrate reductase alpha subunit (*narG*), nitrite reductase (*nirS*), nitric oxide reductase (*norZ*), and nitrous oxide reductase (*nosZ*) genes, nucleotide data sets were downloaded from the NCBI GenBank database (92). Alignments were performed using the “AlignSeqs” command in the DECIPHER “R” package (93). Phylogenetic trees were calculated using neighbor-joining criterion with 1,000 bootstrap tests for every node, and the trees were visualized with the assistance of FigTree v1.4 (http://tree.bio.ed.ac.uk).

### RNA sequencing, filtering, and mapping.

Six biomass samples from within a reactor cycle on operational day 522 (Fig. 1) were collected and immediately processed to determine transcript abundance. RNA was extracted from the samples using an RNeasy kit (Qiagen, Valencia, CA, USA) with a DNase digestion step. RNA integrity and DNA contamination were assessed using an Agilent 2100 Bioanalyzer (Agilent Technologies, Palo Alto, CA, USA). rRNA was removed from 1 μg of total RNA using a Ribo-Zero rRNA removal kit (Bacteria) (Epicentre, Madison, WI, USA). Libraries were generated using a Truseq stranded mRNA sample preparation kit (Illumina, San Diego, CA, USA), according to the manufacturer’s protocol. The libraries were quantified using a next-generation sequencing library quantitative PCR kit (Kapa Biosystems) and run on a Roche LightCycler 480 real-time PCR instrument. The quantified libraries were then prepared for sequencing on an Illumina HiSeq 2000 sequencing platform utilizing a TruSeq paired-end cluster kit (v3) and Illumina’s cBot instrument to generate a clustered flow cell for sequencing. Sequencing of the flow cell was performed on an Illumina HiSeq 2000 sequencer using a TruSeq SBS sequencing kit (v3; 200 cycles), following a 2 × 150 indexed run recipe. Sequence data were deposited at IMG/M under Taxon Object identifiers (IDs) 3300004259, 3300004260, and 3300004621 to 3300004624 (https://genome.jgi.doe.gov/portal/). DNA and RNA-sequencing data sets were obtained in collaboration with the Department of Energy Joint Genome Institute (DOE-JGI).

RNA reads were quality filtered and trimmed with Sickle, and forward and reverse reads were merged using FLASH (v. 1.2.11) (94). rRNA sequences were removed using SortMeRNA and six built-in databases for bacterial, archaeal, and eukaryotic small and large subunits (95). Merged reads that passed filtering were then mapped to UW-LDO-IC using the BBMap suite (96) with the respective default parameters. Read counts were then calculated using HTseq with the “intersection strict” parameter (97). Read counts were normalized by the total number of unmerged reads in the sequencing run, the number of merged reads that remained after rRNA filtering (Table S3), the fraction of merged reads that aligned to the “*Ca*. Accumulibacter phosphatis” UW-LDO-IC genome in each sample (Table S4), and gene length. Numbers of reads mapping to each gene were then converted to log_2_ reads per kilobase per million (RPKM) (98).

### Primer design.

PCR primer sets targeting “*Ca*. Accumulibacter phosphatis” UW-LDO-IC’s genes *nirS*, *narG*, *norZ*, and *nosZ*; the *ccoN* subunit of *cbb3*; and the *ctaD* subunit of *aa3* cytochrome oxidases were designed to quantify expression of these genes in cDNA samples from the reactor. For comparison, primers for the *rpoN* gene were also designed. For primer design, genes from UW-LDO-IC and other published “*Ca*. Accumulibacter phosphatis” genomes were aligned with homologs from bacteria that share relatively high DNA sequence identity with “*Ca*. Accumulibacter phosphatis” (>60% sequence identity). The list of aligned gene sequences was then submitted to DECIPHER’s Design Primers Web tool (99), with results indicating that the sequence of the gene from UW-LDO-IC was the only sequence to be targeted by the primers. Gene homologs from other “*Ca*. Accumulibacter phosphatis” genomes and from non-“*Ca*. Accumulibacter phosphatis” genomes were selected as non-target sequences for which amplification was to be avoided. Additional parameters during primer design performed with DECIPHER included primer lengths ranging from 17 to 26 nucleotides, no more than 2 permutations per primer, a PCR product amplicon length of between 75 and 500 bp, and an annealing temperature of 64°C.

PCR amplification of UW-LDO-IC gene fragments was carried out on extracted genomic DNA from the laboratory-scale SBR in a 25-μl reaction mixture volume with a 400 nM concentration of each of the forward and reverse primers. The PCR program consisted of an initial 10-min denaturation step at 95°C, followed by 30 cycles of 95°C for 30 s, 64°C for 30 s, and 72°C for 30 s and then a final extension step at 72°C for 5 min. The presence and sizes of the amplification products were determined by agarose (2%) gel electrophoresis of the reaction product. The amplified fragments were then purified and cloned using a TOPO TA cloning kit (Invitrogen, CA) according to the manufacturer’s instructions. Fragments were subjected to single-pass Sanger sequencing, and the sequences were aligned to the genome of “*Ca*. Accumulibacter phosphatis” UW-LDO-IC to confirm specificity. In all cases, PCR fragment sequences aligned to the corresponding gene in UW-LDO-IC with an identity level of >97%, whereas the identity levels seen with other “*Ca*. Accumulibacter phosphatis” genomes and other off-target species were <95% and <80%, respectively. The sequences have been deposited in GenBank under BioProject PRJNA482254.

### Quantitative real-time PCR.

cDNA was generated from 500 ng of total RNA, primed by random hexamers (SuperScript II first-strand synthesis system; Invitrogen, Carlsbad, CA, USA). The reaction was terminated by incubation at 85°C for 5 min, and RNase H treatment was performed to degrade RNA in RNA:DNA hybrids. Subsequently, 4 μl of 10× diluted cDNA was applied as the template in qPCR. All quantifications were performed in triplicate. The qPCR was conducted on a LightCycler 480 system (Roche, Switzerland) using iQ SYBR green Supermix (Bio-Rad) with a total reaction mixture volume of 20 μl. All qPCR programs consisted of an initial 3-min denaturation at 95°C, followed by 45 cycles of denaturing at 95°C for 30 s, 64°C for 30 s, and 72°C for 30 s. RNA samples without reverse transcription were used as no-RT (reverse transcription) controls to evaluate DNA contamination for all primers tested. The relative fold change of target gene expression between samples was quantified using “*Ca*. Accumulibacter phosphatis” *rpoN* as a reference gene, since this is a constitutively expressed gene with a constant expression level.

### Operons and upstream motif identification.

*De novo* motif detection analysis was conducted on the intergenic regions upstream of the *nar*, *nir*, *nor*, *nos*, *aa3*, and *cbb3* operons of “*Ca*. Accumulibacter phosphatis” UW-LDO-IC using MEME (100). FNR motif sites were further identified in both strands of other “*Ca*. Accumulibacter phosphatis” genomes using the FIMO tool (101) (*P* values, <1 × 10^−5^). Search was limited to the promoter region, represented by the 300-bp intergenic region upstream of the transcriptional start site, of all protein-coding sequences annotated with MetaPathways (89).

Putative operons were determined using a previously described set of criteria (49); that is, each operon enclosed adjacent genes with the same orientation, coexpressed with a minimum Pearce correlation coefficient of 0.7, and an intergenic region between genes of 1,000 bp or fewer.

### Accession number(s).

The sequencing data determined in this work are available under BioProject PRJNA482250, PRJNA482254, and PRJNA322674. The metagenomic assembly and final “*Ca*. Accumulibacter phosphatis” bin can be found under GenBank accession number QPGA00000000. Sequence data were deposited at IMG/M under Taxon Object IDs 3300004259, 3300004260, and 3300004621 to 3300004624 (https://genome.jgi.doe.gov/portal/).
