# Supplementary material for: Integrated Omic Analyses Provide Evidence that a “Candidatus Accumulibacter phosphatis” Strain Performs Denitrification under Microaerobic Conditions
Source: mSystems. 2019 Jan 15;4(1):e00193-18. doi: 10.1128/mSystems.00193-18 (PMC6446978; doi:10.1128/mSystems.00193-18)
Supplement: TABLE S1 [file mSystems.00193-18-st001.docx]

| Bin Id | Lowest Assigned Taxonomy Rank | GC (%) | Genome size (Mbp) | # Scaffolds | N50 | Completeness (%) | Redundancy (%) | Strain heterogeneity (%) | Mean Coverage 522-day |
| --- | --- | --- | --- | --- | --- | --- | --- | --- | --- |
|  |  |  |  |  |  |  |  |  |  |
| Bin 001 | Unresolved | 60.3 | 0.15 | 5 | 133,274 | 0.0 | 0.0 | 0.0 | 1.75 |
| Bin 002 | Bacteria; Proteobacteria; Gammaproteobacteria; Chromatiales; Ectothiorhodospiraceae | 58.1 | 1.88 | 95 | 32,415 | 70.1 | 0.5 | 0.0 | 7.93 |
| Bin 003 | Bacteria; Proteobacteria; Gammaproteobacteria | 57.9 | 1.19 | 104 | 19,456 | 18.9 | 0.0 | 0.0 | 4.67 |
| Bin 004 | Unresolved | 61.4 | 0.46 | 71 | 9,751 | 0.0 | 0.0 | 0.0 | 1.62 |
| Bin 005 | Unresolved | 53.2 | 0.44 | 90 | 8,194 | 0.0 | 0.0 | 0.0 | 1.34 |
| Bin 006 | Unresolved | 60.8 | 0.44 | 117 | 5,609 | 4.2 | 0.0 | 0.0 | 1.26 |
| Bin 007 | Unresolved | 55.0 | 0.32 | 59 | 10,982 | 0.0 | 0.0 | 0.0 | 0.52 |
| Bin 008 | Bacteria; Verrucomicrobia | 62.6 | 3.88 | 26 | 501,618 | 96.8 | 3.4 | 0.0 | 5.61 |
| Bin 009 | Unresolved | 45.2 | 0.23 | 59 | 5,784 | 0.0 | 0.0 | 0.0 | 0.26 |
| Bin 010 | Bacteria; Bacteroidetes; Sphingobacteriia; Sphingobacteriales; Saprospiraceae; Lewinella | 55.6 | 2.21 | 24 | 176,250 | 39.7 | 0.0 | 0.0 | 2.28 |
| Bin 011 | Bacteria; Bacteroidetes; Sphingobacteriia; Sphingobacteriales; Saprospiraceae; Lewinella | 54.7 | 2.10 | 23 | 127,440 | 31.0 | 0.0 | 0.0 | 2.23 |
| Bin 012 | Bacteria | 62.0 | 2.81 | 50 | 2,659,120 | 94.9 | 1.9 | 0.0 | 2.91 |
| Bin 013 | Bacteria; Proteobacteria; Alphaproteobacteria; Rhodobacterales; Hyphomonadaceae | 45.3 | 2.85 | 67 | 182,623 | 97.6 | 1.0 | 25.0 | 1.97 |
| Bin 014 | Bacteria; Proteobacteria | 58.4 | 1.44 | 299 | 7,814 | 1.8 | 0.0 | 0.0 | 0.92 |
| Bin 015 | Bacteria; Chloroflexi; Caldilineae; Caldilineales; Caldilineaceae; Caldilinea | 64.3 | 5.77 | 307 | 38,066 | 92.3 | 1.1 | 0.0 | 3.36 |
| Bin 016 | Bacteria; Proteobacteria; Alphaproteobacteria; Rhodospirillales; Rhodospirillaceae | 67.7 | 5.05 | 273 | 46,256 | 98.3 | 37.7 | 0.9 | 2.76 |
| Bin 017 | Bacteria; Bacteroidetes; Flavobacteriia; Flavobacteriales; Cryomorphaceae; Fluviicola; Fluviicola | 60.8 | 3.85 | 87 | 78,231 | 98.9 | 0.2 | 0.0 | 1.92 |
| Bin 018 | Bacteria; Proteobacteria; Betaproteobacteria; Rhodocyclales; Rhodocyclaceae; Dechloromonas | 63.8 | 2.65 | 439 | 11,996 | 47.8 | 5.5 | 54.2 | 1.20 |
| Bin 019 | Bacteria; Bacteroidetes; Cytophagia; Cytophagales; Flammeovirgaceae; Fulvivirga | 42.5 | 4.32 | 79 | 1,137,187 | 98.8 | 2.1 | 11.1 | 1.85 |
| Bin 020 | Bacteria; Proteobacteria; Alphaproteobacteria; Rhodospirillales; Rhodospirillaceae | 69.9 | 2.47 | 54 | 101,360 | 75.2 | 0.0 | 0.0 | 1.00 |
| Bin 021 | Bacteria; Proteobacteria; Alphaproteobacteria; Rhizobiales; Beijerinckiaceae; Beijerinckia | 67.0 | 3.41 | 33 | 246,859 | 99.1 | 1.0 | 0.0 | 1.26 |
| Bin 022 | Bacteria; Proteobacteria; Alphaproteobacteria; Rhodospirillales | 68.9 | 1.25 | 258 | 8,604 | 17.2 | 0.0 | 0.0 | 0.43 |
| Bin 023 | Bacteria | 57.0 | 3.53 | 178 | 329,776 | 95.4 | 2.8 | 0.0 | 1.10 |
| Bin 024 | Bacteria | 34.8 | 6.28 | 239 | 74,362 | 94.6 | 2.0 | 0.0 | 1.74 |
| Bin 025 | Bacteria; Proteobacteria; Gammaproteobacteria; Chromatiales | 57.4 | 5.28 | 158 | 369,154 | 100.0 | 6.9 | 94.1 | 1.30 |
| Bin 026 | Bacteria; Bacteroidetes; Flavobacteriia; Bacteroidetes | 46.3 | 4.59 | 94 | 88,733 | 97.0 | 2.5 | 0.0 | 1.07 |
| Bin 027 | Bacteria; Proteobacteria; Alphaproteobacteria; Caulobacterales; Caulobacteraceae; Brevundimonas | 66.6 | 2.70 | 220 | 22,667 | 99.0 | 7.0 | 78.3 | 0.53 |
| Bin 028 | Bacteria; Proteobacteria; Alphaproteobacteria; Rhizobiales | 67.2 | 3.50 | 590 | 11,319 | 75.9 | 10.3 | 20.0 | 0.67 |
| Bin 029 | Bacteria; Bacteroidetes; Cytophagia; Cytophagales; Flammeovirgaceae; Fulvivirga | 42.1 | 4.68 | 90 | 246,705 | 99.7 | 1.6 | 0.0 | 0.77 |
| Bin 030 | Bacteria; Proteobacteria; Gammaproteobacteria | 56.6 | 3.64 | 70 | 129,915 | 93.1 | 29.7 | 34.8 | 0.62 |
| Bin 031.1 | Bacteria; Proteobacteria; Gammaproteobacteria | 59.6 | 2.81 | 84 | 166,389 | 72.7 | 2.3 | 0.0 | 0.44 |

Completeness, redundancy and strain heterogeneity were assessed with CHECKM 0.7.1, according to the presence of 43 single-copy reference gene. Bins designated as “Bin XXX.X” were generated using anvi’o (*See Methods*).

| Bin Id | Lowest Assigned Taxonomy Rank | GC (%) | Genome size (Mbp) | # Scaffolds | N50 | Completeness (%) | Redundancy (%) | Strain heterogeneity (%) | Mean Coverage 522-day |
| --- | --- | --- | --- | --- | --- | --- | --- | --- | --- |
|  |  |  |  |  |  |  |  |  |  |
| Bin 031.2 | Bacteria; Proteobacteria; Gammaproteobacteria; Chromatiales | 59.4 | 1.84 | 256 | 14,316 | 28.7 | 4.5 | 25.9 | 0.28 |
| Bin 032 | Bacteria; Ignavibacteriae; Ignavibacteria; Ignavibacteriales; Ignavibacteriaceae; Ignavibacterium | 39.2 | 4.42 | 103 | 113,436 | 98.3 | 0.6 | 0.0 | 0.68 |
| Bin 033 | Bacteria; Proteobacteria; Alphaproteobacteria; Rhizobiales | 65.4 | 4.46 | 333 | 153,360 | 98.3 | 25.5 | 34.2 | 0.63 |
| Bin 034 | Bacteria; Proteobacteria; Gammaproteobacteria | 62.3 | 4.47 | 586 | 24,795 | 87.9 | 40.3 | 14.4 | 0.64 |
| Bin 035 | Bacteria; Verrucomicrobia; Verrucomicrobiae; Verrucomicrobiales; Verrucomicrobiaceae; Verrucomicrobium | 58.7 | 8.63 | 310 | 173,586 | 99.3 | 8.8 | 8.7 | 1.03 |
| Bin 036 | Bacteria; Proteobacteria; Alphaproteobacteria; Rhodospirillales; Rhodospirillaceae | 55.6 | 2.96 | 213 | 37,364 | 96.8 | 11.3 | 0.0 | 0.35 |
| Bin 037 | Bacteria; Proteobacteria; Alphaproteobacteria; Rhizobiales; Methylobacteriaceae | 63.0 | 4.94 | 243 | 569,899 | 98.3 | 37.0 | 16.8 | 0.59 |
| Bin 038.2 | Bacteria; Proteobacteria; Betaproteobacteria; Rhodocyclales; Rhodocyclaceae; Thauera | 69.6 | 1.90 | 252 | 12,628 | 63.2 | 1.8 | 100.0 | 0.30 |
| Bin 038.3 | Bacteria; Proteobacteria; Betaproteobacteria; Rhodocyclales; Rhodocyclaceae; Thauera | 70.7 | 2.69 | 589 | 7,493 | 22.3 | 7.0 | 50.0 | 0.27 |
| Bin 039 | Bacteria; Proteobacteria; Betaproteobacteria; Gallionellales; Gallionellaceae; Gallionella | 49.6 | 2.19 | 536 | 5,330 | 85.7 | 5.0 | 84.2 | 0.26 |
| Bin 040 | Bacteria; Verrucomicrobia; Verrucomicrobiae; Verrucomicrobiales; Verrucomicrobiaceae; Verrucomicrobium | 60.8 | 7.21 | 277 | 52,427 | 93.6 | 6.9 | 65.2 | 0.83 |
| Bin 041 | Bacteria; Proteobacteria; Alphaproteobacteria; Rhodospirillales; Rhodospirillaceae; Rhodospirillum | 64.9 | 3.95 | 176 | 197,681 | 100.0 | 18.2 | 11.1 | 0.43 |
| Bin 042 | Bacteria; Proteobacteria; Deltaproteobacteria | 33.1 | 3.29 | 61 | 629,816 | 96.1 | 0.7 | 0.0 | 0.34 |
| Bin 043 | Bacteria | 51.5 | 3.15 | 90 | 221,518 | 95.3 | 4.8 | 66.7 | 0.32 |
| Bin 044 | Bacteria; Actinobacteria; Actinobacteria; Acidimicrobiales | 72.7 | 5.44 | 558 | 294,835 | 99.2 | 13.3 | 0.0 | 0.54 |
| Bin 045 | Bacteria; Proteobacteria; Betaproteobacteria; Nitrosomonadales; Nitrosomonadaceae; Nitrosomonas | 49.4 | 2.86 | 296 | 16,241 | 88.3 | 2.7 | 64.3 | 0.28 |
| Bin 046 | Bacteria; Proteobacteria; Betaproteobacteria; Rhodocyclales; Rhodocyclaceae; Accumulibacter phosphatis | 62.6 | 6.60 | 666 | 23,909 | 94.8 | 28.9 | 20.3 | 0.64 |
| Bin 047 | Bacteria; Proteobacteria; Gammaproteobacteria; Chromatiales | 63.9 | 4.39 | 782 | 13,343 | 79.7 | 21.9 | 27.9 | 0.39 |
| Bin 048 | Bacteria; Proteobacteria; Alphaproteobacteria; Rhodospirillales; Rhodospirillaceae | 68.6 | 4.24 | 262 | 119,328 | 90.6 | 8.6 | 0.0 | 0.39 |
| Bin 049.1 | Bacteria; Proteobacteria; Betaproteobacteria; Rhodocyclales; Rhodocyclaceae; Thauera | 67.8 | 0.83 | 180 | 8,572 | 9.3 | 2.7 | 63.2 | 0.07 |
| Bin 049.2 | Bacteria; Proteobacteria; Betaproteobacteria; Rhodocyclales; Rhodocyclaceae; Thauera | 67.5 | 2.18 | 213 | 23,756 | 34.2 | 3.5 | 100.0 | 0.19 |
| Bin 049.3 | Bacteria; Proteobacteria; Betaproteobacteria; Rhodocyclales; Rhodocyclaceae; Thauera | 68.1 | 1.50 | 225 | 14,219 | 40.4 | 3.5 | 100.0 | 0.14 |
| Bin 049.4 | Bacteria; Proteobacteria; Betaproteobacteria; Rhodocyclales; Rhodocyclaceae; Thauera | 67.5 | 1.09 | 316 | 4,763 | 31.6 | 0.9 | 30.8 | 0.13 |
| Bin 050 | Bacteria; Proteobacteria; Alphaproteobacteria; Rhodobacterales; Rhodobacteraceae; Rhodobacter | 67.1 | 3.68 | 260 | 32,433 | 96.1 | 5.4 | 19.2 | 0.30 |
| Bin 051 | Bacteria; Bacteroidetes; Cytophagia | 44.3 | 4.46 | 211 | 48,531 | 89.1 | 1.2 | 0.0 | 0.35 |
| Bin 052 | Bacteria; Planctomycetes; Phycisphaerae; Phycisphaerales; Phycisphaeraceae; Phycisphaera | 63.8 | 3.53 | 138 | 136,152 | 95.5 | 1.9 | 0.0 | 0.27 |
| Bin 053 | Bacteria; Chloroflexi; Anaerolineae; Anaerolineales; Anaerolineaceae; Anaerolinea | 53.8 | 6.46 | 1,214 | 9,633 | 90.2 | 27.5 | 15.2 | 0.50 |
| Bin 054 | Bacteria; Verrucomicrobia; Verrucomicrobiae; Verrucomicrobiales; Verrucomicrobiaceae | 62.3 | 7.57 | 1,074 | 19,131 | 99.3 | 16.9 | 19.1 | 0.55 |
| Bin 055.1 | Bacteria; Bacteroidetes; Sphingobacteriia; Sphingobacteriales; Saprospiraceae | 52.6 | 1.50 | 721 | 2,272 | 29.3 | 1.7 | 100.0 | 0.06 |
| Bin 055.2 | Bacteria; Bacteroidetes; Sphingobacteriia; Sphingobacteriales; Saprospiraceae | 50.2 | 6.61 | 293 | 46,761 | 99.5 | 2.5 | 16.7 | 0.56 |
| Bin 056 | Bacteria; Proteobacteria; Gammaproteobacteria | 59.1 | 2.25 | 419 | 10,463 | 32.8 | 4.9 | 50.0 | 0.17 |

Completeness, redundancy and strain heterogeneity were assessed with CHECKM 0.7.1, according to the presence of 43 single-copy reference gene. Bins designated as “bin.XXX.X” were generated using anvi’o (*See Methods*).

| Bin Id | Lowest Assigned Taxonomy Rank | GC (%) | Genome size (Mbp) | # Scaffolds | N50 | Completeness (%) | Redundancy (%) | Strain heterogeneity (%) | Mean Coverage 522-day |
| --- | --- | --- | --- | --- | --- | --- | --- | --- | --- |
|  |  |  |  |  |  |  |  |  |  |
| Bin 057 | Bacteria; Proteobacteria; Gammaproteobacteria; Chromatiales | 57.9 | 2.43 | 376 | 10,849 | 61.3 | 15.5 | 84.0 | 0.17 |
| Bin 058 | Bacteria; Bacteroidetes; Sphingobacteriia; Sphingobacteriales | 40.2 | 3.39 | 132 | 75,779 | 97.8 | 1.0 | 0.0 | 0.23 |
| Bin 059 | Bacteria; Proteobacteria; Gammaproteobacteria; Chromatiales | 60.8 | 4.07 | 834 | 9,247 | 60.3 | 30.2 | 43.5 | 0.27 |
| Bin 060 | Bacteria | 70.1 | 5.83 | 239 | 51,428 | 97.9 | 6.8 | 9.1 | 0.38 |
| Bin 061 | Bacteria; Proteobacteria; Betaproteobacteria; Burkholderiales | 71.0 | 6.75 | 1,221 | 34,227 | 86.7 | 15.4 | 5.6 | 0.44 |
| Bin 062 | Bacteria; Actinobacteria; Actinobacteria; Acidimicrobiales; Acidimicrobiaceae | 41.3 | 2.11 | 148 | 91,911 | 99.2 | 11.0 | 0.0 | 0.14 |
| Bin 063 | Bacteria; Planctomycetes; Planctomycetia; Planctomycetales; Planctomycetaceae; Planctomyces | 56.4 | 7.39 | 1,206 | 11,128 | 95.5 | 2.3 | 100.0 | 0.47 |
| Bin 064 | Bacteria | 47.4 | 2.14 | 842 | 2,929 | 15.2 | 0.8 | 0.0 | 0.13 |
| Bin 065 | Bacteria; Verrucomicrobia; Verrucomicrobiae; Verrucomicrobiales; Verrucomicrobiaceae; Verrucomicrobium | 60.8 | 6.31 | 1,166 | 10,868 | 83.5 | 15.6 | 30.6 | 0.36 |
| Bin 066 | Unresolved | 59.7 | 6.64 | 1,127 | 21,161 | 100.0 | 43.7 | 2.7 | 0.38 |
| Bin 067 | Bacteria; Bacteroidetes; Sphingobacteriia; Sphingobacteriales; Saprospiraceae; Lewinella | 39.1 | 7.07 | 982 | 16,257 | 100.0 | 55.3 | 0.0 | 0.40 |
| Bin 068 | Bacteria; Chloroflexi | 66.0 | 5.63 | 568 | 17,767 | 97.3 | 8.8 | 25.0 | 0.31 |
| Bin 069 | Bacteria | 62.3 | 2.13 | 378 | 10,706 | 87.9 | 25.9 | 2.9 | 0.12 |
| Bin 070 | Bacteria; Bacteroidetes; Sphingobacteriia; Sphingobacteriales | 37.0 | 4.26 | 771 | 15,497 | 98.7 | 9.3 | 9.5 | 0.23 |
| Bin 071 | Bacteria; Proteobacteria; Alphaproteobacteria; Caulobacterales | 66.4 | 3.39 | 972 | 4,531 | 49.8 | 34.8 | 53.9 | 0.20 |
| Bin 072 | Bacteria; Bacteroidetes; Sphingobacteriia; Sphingobacteriales | 46.7 | 4.19 | 1,020 | 6,192 | 93.4 | 5.6 | 57.1 | 0.23 |
| Bin 073 | Bacteria; Proteobacteria; Alphaproteobacteria | 41.8 | 3.14 | 983 | 4,183 | 82.3 | 17.1 | 3.2 | 0.17 |
| Bin 074 | Bacteria; Bacteroidetes; Sphingobacteriia; Sphingobacteriales; Sphingobacteriaceae; Niabella | 40.4 | 6.78 | 1,798 | 10,294 | 99.5 | 53.7 | 5.5 | 0.35 |
| Bin 075 | Bacteria; Bacteroidetes; Cytophagia; Cytophagales; Flammeovirgaceae; Fulvivirga | 42.9 | 3.84 | 788 | 7,441 | 91.4 | 23.8 | 8.9 | 0.20 |
| Bin 076 | Bacteria; Actinobacteria; Actinobacteria | 40.7 | 2.10 | 513 | 7,103 | 96.6 | 31.6 | 1.7 | 0.09 |
| Bin 077 | Bacteria | 57.3 | 3.41 | 701 | 10,279 | 91.7 | 15.3 | 5.1 | 0.16 |
| Bin 078 | Bacteria; Bacteroidetes; Flavobacteriia; Flavobacteriales; Flavobacteriaceae | 35.6 | 6.32 | 1,559 | 7,104 | 92.2 | 46.5 | 4.0 | 0.29 |
| Bin 079 | Bacteria; Proteobacteria; Alphaproteobacteria; Caulobacterales; Caulobacteraceae; Brevundimonas | 65.6 | 4.06 | 1,406 | 3,852 | 71.6 | 55.7 | 26.4 | 0.18 |
| Bin 080 | Bacteria; Proteobacteria; Alphaproteobacteria; Rhizobiales | 69.0 | 3.89 | 1,574 | 2,847 | 46.0 | 28.1 | 25.0 | 0.18 |
| Bin 081 | Bacteria; Proteobacteria; Alphaproteobacteria; Caulobacterales; Caulobacteraceae; Brevundimonas | 69.0 | 6.20 | 2,724 | 2,552 | 77.7 | 65.5 | 16.7 | 0.30 |
| Bin 082 | Bacteria; Proteobacteria; Alphaproteobacteria; Rhizobiales; Phyllobacteriaceae | 67.5 | 4.21 | 1,806 | 2,664 | 63.6 | 32.9 | 18.2 | 0.19 |
| Bin 083 | Bacteria; Proteobacteria; Alphaproteobacteria; Rhizobiales; Phyllobacteriaceae | 65.3 | 1.33 | 566 | 2,758 | 32.7 | 5.5 | 37.5 | 0.06 |
| Bin 084 | Bacteria; Proteobacteria; Alphaproteobacteria; Rhizobiales; Phyllobacteriaceae | 64.0 | 1.19 | 415 | 4,346 | 19.1 | 2.6 | 12.8 | 0.05 |
| Bin 085 | Bacteria; Proteobacteria; Alphaproteobacteria; Rhizobiales; Hyphomicrobiaceae; Hyphomicrobium | 66.0 | 4.85 | 1,315 | 5,676 | 49.7 | 24.1 | 0.0 | 0.21 |
| Bin 086 | Bacteria; Bacteroidetes | 48.0 | 1.27 | 633 | 2,002 | 13.3 | 1.0 | 0.0 | 0.06 |
| Bin 087 | Bacteria; Chloroflexi; Caldilineae; Caldilineales; Caldilineaceae; Caldilinea | 59.3 | 6.80 | 1,633 | 5,903 | 84.0 | 6.6 | 12.5 | 0.29 |

Completeness, redundancy and strain heterogeneity were assessed with CHECKM 0.7.1, according to the presence of 43 single-copy reference gene

Bins designated as “bin.XXX.X” were generated using anvi’o (*See Methods*).

| Bin Id | Lowest Assigned Taxonomy Rank | GC (%) | Genome size (Mbp) | # Scaffolds | N50 | Completeness (%) | Redundancy (%) | Strain heterogeneity (%) | Mean Coverage 522-day |
| --- | --- | --- | --- | --- | --- | --- | --- | --- | --- |
|  |  |  |  |  |  |  |  |  |  |
| Bin 088 | Bacteria; Bacteroidetes | 44.1 | 1.43 | 561 | 3,129 | 6.0 | 0.0 | 0.0 | 0.06 |
| Bin 089 | Bacteria; Proteobacteria; Deltaproteobacteria; Bdellovibrionales; Bdellovibrionaceae; Bdellovibrio | 53.8 | 4.56 | 1,371 | 6,984 | 94.7 | 64.0 | 2.9 | 0.19 |
| Bin 090 | Bacteria; Bacteroidetes | 43.8 | 2.82 | 819 | 4,720 | 85.8 | 9.9 | 3.6 | 0.11 |
| Bin 091 | Bacteria; Proteobacteria; Betaproteobacteria; Burkholderiales | 67.0 | 8.09 | 3,579 | 2,391 | 65.5 | 59.5 | 3.4 | 0.32 |
| Bin 092 | Bacteria; Proteobacteria; Alphaproteobacteria; Rhizobiales | 65.8 | 0.64 | 333 | 1,995 | 21.0 | 12.3 | 0.0 | 0.03 |
| Bin 093 | Bacteria; Proteobacteria; Alphaproteobacteria; Rhizobiales | 65.5 | 0.51 | 290 | 1,714 | 23.3 | 23.5 | 9.4 | 0.02 |
| Bin 094 | Bacteria; Proteobacteria; Betaproteobacteria; Burkholderiales | 72.2 | 6.56 | 2,711 | 2,926 | 62.1 | 18.1 | 26.7 | 0.26 |
| Bin 095 | Bacteria; Proteobacteria; Deltaproteobacteria; Bdellovibrionales; Bdellovibrionaceae; Bdellovibrio | 38.3 | 2.98 | 1,141 | 2,768 | 80.5 | 37.8 | 1.9 | 0.11 |
| Bin 096 | Bacteria | 57.7 | 3.88 | 1,960 | 1,962 | 49.0 | 39.4 | 2.8 | 0.15 |
| Bin 097.1 | Bacteria; Proteobacteria; Betaproteobacteria | 62.9 | 1.23 | 651 | 1,962 | 27.1 | 1.7 | 14.3 | 0.05 |
| Bin 097.2 | Bacteria; Proteobacteria; Gammaproteobacteria | 63.4 | 3.13 | 1,645 | 1,941 | 24.3 | 3.4 | 0.0 | 0.11 |
| Bin 097.3 | Bacteria; Proteobacteria; Gammaproteobacteria | 63.2 | 2.54 | 1,101 | 2,687 | 51.5 | 9.6 | 0.0 | 0.09 |
| Bin 097.4 | Bacteria; Proteobacteria; Betaproteobacteria; Rhodocyclales; Rhodocyclaceae; Accumulibacter phosphatis | 63.5 | 0.93 | 274 | 4,779 | 27.0 | 4.3 | 33.3 | 0.04 |
| Bin 098 | Bacteria; Planctomycetes; Phycisphaerae; Phycisphaerales; Phycisphaeraceae; Phycisphaera | 69.6 | 6.72 | 2,642 | 3,009 | 88.7 | 56.8 | 1.2 | 0.25 |
| Bin 099 | Bacteria; Proteobacteria; Alphaproteobacteria; Rhodospirillales | 70.3 | 4.89 | 2,736 | 1,821 | 62.2 | 51.7 | 6.3 | 0.18 |
| Bin 100 | Bacteria | 45.7 | 1.02 | 466 | 2,321 | 52.7 | 15.3 | 2.7 | 0.04 |
| Bin 101 | Bacteria; Proteobacteria; Alphaproteobacteria; Rhodobacterales; Rhodobacteraceae; Rhodobacter | 65.0 | 3.12 | 1,836 | 1,690 | 43.8 | 15.7 | 26.7 | 0.12 |
| Bin 102 | Bacteria; Proteobacteria; Alphaproteobacteria; Rhodobacterales; Rhodobacteraceae; Rhodobacter | 69.2 | 5.11 | 2,623 | 2,055 | 61.2 | 64.7 | 9.5 | 0.18 |
| Bin 103 | Bacteria; Proteobacteria; Deltaproteobacteria; Myxococcales; Polyangiaceae; Sorangium | 70.5 | 7.09 | 2,712 | 3,101 | 82.2 | 7.1 | 15.4 | 0.25 |
| Bin 104 | Bacteria | 57.0 | 7.55 | 3,857 | 2,060 | 62.3 | 22.4 | 2.0 | 0.25 |
| Bin 105 | Bacteria; Proteobacteria; Alphaproteobacteria; Rhizobiales | 71.6 | 2.94 | 1,919 | 1,471 | 42.2 | 16.4 | 12.5 | 0.10 |
| Bin 106 | Bacteria; Proteobacteria; Alphaproteobacteria; Rhodobacterales; Hyphomonadaceae | 64.4 | 3.41 | 1,360 | 3,040 | 82.2 | 13.7 | 7.6 | 0.12 |
| Bin 107 | Bacteria; Proteobacteria; Alphaproteobacteria; Rhizobiales | 63.0 | 3.91 | 1,954 | 2,128 | 67.2 | 35.0 | 4.7 | 0.14 |
| Bin 108 | Bacteria; Proteobacteria; Alphaproteobacteria; Rhodobacterales; Rhodobacteraceae | 66.2 | 3.43 | 1,770 | 1,972 | 59.1 | 32.4 | 24.9 | 0.12 |
| Bin 109 | Bacteria; Proteobacteria; Alphaproteobacteria; Rhizobiales | 64.7 | 3.68 | 1,845 | 2,106 | 65.5 | 23.3 | 0.0 | 0.13 |
| Bin 110 | Bacteria; Proteobacteria; Alphaproteobacteria; Rickettsiales | 40.6 | 1.48 | 567 | 3,037 | 88.2 | 3.7 | 50.0 | 0.05 |
| Bin 111 | Bacteria; Bacteroidetes; Bacteroidia; Bacteroidales | 43.6 | 2.47 | 1,462 | 1,718 | 60.9 | 5.0 | 15.4 | 0.08 |
| Bin 112 | Bacteria; Bacteroidetes; Sphingobacteriia; Sphingobacteriales | 43.2 | 2.51 | 1,144 | 2,465 | 68.4 | 4.9 | 26.7 | 0.08 |
| Bin 113 | Bacteria | 51.5 | 8.28 | 4,346 | 1,986 | 80.8 | 38.7 | 1.6 | 0.26 |
| Bin 114 | Bacteria; Proteobacteria; Alphaproteobacteria | 62.2 | 3.72 | 2,141 | 1,661 | 38.7 | 25.2 | 1.9 | 0.12 |
| Bin 115 | Bacteria; Proteobacteria; Alphaproteobacteria; Rhizobiales | 58.5 | 2.35 | 1,228 | 1,997 | 54.5 | 5.9 | 7.3 | 0.07 |

Completeness, redundancy and strain heterogeneity were assessed with CHECKM 0.7.1, according to the presence of 43 single-copy reference gene

Bins designated as “bin.XXX.X” were generated using anvi’o (*See Methods*).

| Bin Id | Lowest Assigned Taxonomy Rank | GC (%) | Genome size (Mbp) | # Scaffolds | N50 | Completeness (%) | Redundancy (%) | Strain heterogeneity (%) | Mean Coverage 522-day |
| --- | --- | --- | --- | --- | --- | --- | --- | --- | --- |
|  |  |  |  |  |  |  |  |  |  |
| Bin 116 | Bacteria; Bacteroidetes | 66.8 | 2.51 | 1,220 | 2,273 | 65.0 | 8.5 | 4.0 | 0.08 |
| Bin 117 | Bacteria; Bacteroidetes; Sphingobacteriia; Sphingobacteriales | 30.6 | 2.17 | 1,293 | 1,580 | 53.1 | 4.7 | 0.0 | 0.07 |
| Bin 118 | Bacteria; Planctomycetes; Planctomycetia; Planctomycetales; Planctomycetaceae; Planctomyces | 58.2 | 4.64 | 2,302 | 2,163 | 69.8 | 3.1 | 14.3 | 0.15 |
| Bin 119.1 | Bacteria | 66.5 | 0.67 | 350 | 1,901 | 6.6 | 1.0 | 0.0 | 0.02 |
| Bin 119.2 | Bacteria; Planctomycetes; Phycisphaerae; Phycisphaerales; Phycisphaeraceae; Phycisphaera | 66.4 | 1.57 | 797 | 1,955 | 21.9 | 3.0 | 10.0 | 0.05 |
| Bin 119.3 | Bacteria | 66.5 | 1.66 | 972 | 1,716 | 24.7 | 0.1 | 0.0 | 0.05 |
| Bin 119.4 | Bacteria; Proteobacteria | 66.9 | 0.99 | 663 | 1,451 | 10.2 | 0.9 | 0.0 | 0.02 |
| Bin 120 | Bacteria; Chloroflexi; Caldilineae; Caldilineales; Caldilineaceae; Caldilinea | 60.8 | 4.35 | 2,538 | 1,728 | 59.3 | 16.4 | 0.0 | 0.13 |
| Bin 121 | Bacteria | 69.7 | 4.85 | 2,799 | 1,765 | 57.7 | 17.4 | 0.0 | 0.15 |
| Bin 122 | Bacteria; Proteobacteria; Gammaproteobacteria; Chromatiales; Chromatiaceae | 66.7 | 3.36 | 2,112 | 1,600 | 54.4 | 18.6 | 33.3 | 0.10 |
| Bin 123 | Bacteria; Acidobacteria | 51.6 | 2.14 | 1,195 | 1,867 | 46.3 | 2.8 | 22.2 | 0.06 |
| Bin 124 | Bacteria | 63.3 | 3.46 | 2,223 | 1,533 | 49.3 | 15.8 | 0.0 | 0.10 |

Completeness, redundancy and strain heterogeneity were assessed with CHECKM 0.7.1, according to the presence of 43 single-copy reference gene

Bins designated as “bin.XXX.X” were generated using anvi’o (*See Materials and Method*
