# Supplementary material for: Integrated Omic Analyses Provide Evidence that a “Candidatus Accumulibacter phosphatis” Strain Performs Denitrification under Microaerobic Conditions
Source: mSystems. 2019 Jan 15;4(1):e00193-18. doi: 10.1128/mSystems.00193-18 (PMC6446978; doi:10.1128/mSystems.00193-18)
Supplement: TABLE S2 [file mSystems.00193-18-st002.docx]

| Refinement step | GC (%) | Genome size (Mbp) | Gene count | # Scaffolds | N50 | Completeness (%) | Redundancy (%) | Strain heterogeneity (%) |
| --- | --- | --- | --- | --- | --- | --- | --- | --- |
|  |  |  |  |  |  |  |  |  |
| MaxBin (bin.046) | 62.60 | 6.60 | 6,291 | 666 | 23,909 | 94.8 | 28.94 | 20.29 |
| Anvio decontamination | 62.49 | 4.55 | 4,097 | 239 | 36,358 | 94.8 | 0.84 | 100.00 |
| Nanopore Links scaffolding | 62.49 | 4.61 | 4,074 | 120 | 85,302 | 94.8 | 0.68 | 100.00 |
| Gapcloser | 62.48 | 4.70 | 4,166 | 120 | 85,321 | 95.2 | 0.68 | 100.00 |

Completeness, redundancy and strain heterogeneity were assessed with CHECKM 0.7.1, according to the presence of 43 single-copy reference genes.
