# Supplementary material for: Integrated Omic Analyses Provide Evidence that a “Candidatus Accumulibacter phosphatis” Strain Performs Denitrification under Microaerobic Conditions
Source: mSystems. 2019 Jan 15;4(1):e00193-18. doi: 10.1128/mSystems.00193-18 (PMC6446978; doi:10.1128/mSystems.00193-18)
Supplement: TABLE S3 [file mSystems.00193-18-st003.docx]

| Sample | TimeA Anaerobic-10min | TimeB Anaerobic-32min | TimeC Aerobic-0min | TimeD Aerobic-22min | TimeE Aerobic-52min | TimeF Aerobic-292min |
| --- | --- | --- | --- | --- | --- | --- |
| Total Reads | 317,752,258 | 289,487,298 | 279,849,682 | 272,986,806 | 271,430,590 | 286,971,580 |
| Normalization  Factor from Total Reads | 1.17 | 1.07 | 1.03 | 1.01 | 1.00 | 1.06 |
| Quality Reads | 312,696,898 | 285,415,418 | 275,951,004 | 270,309,846 | 268,730,228 | 283,344,794 |
| Merged Reads | 98,190,636 | 88,219,624 | 83,934,087 | 83,755,245 | 84,265,579 | 83,962,296 |
| rRNA Sequences | 27,374,143 | 21,015,585 | 22,055,875 | 17,613,784 | 15,900,468 | 21,372,211 |
| non rRNA Sequences | 70,816,493 | 67,204,039 | 61,878,212 | 66,141,461 | 68,365,111 | 62,590,085 |
| Normalization  Factor from Filtered Reads | 1.14 | 1.09 | 1.00 | 1.07 | 1.10 | 1.01 |
