# Supplementary material for: Integrated Omic Analyses Provide Evidence that a “Candidatus Accumulibacter phosphatis” Strain Performs Denitrification under Microaerobic Conditions
Source: mSystems. 2019 Jan 15;4(1):e00193-18. doi: 10.1128/mSystems.00193-18 (PMC6446978; doi:10.1128/mSystems.00193-18)
Supplement: TABLE S4 [file mSystems.00193-18-st004.docx]

| **Sample** | **TimeA Anaerobic-10min** | | **TimeB Anaerobic-32min** | | **TimeC Aerobic-0min** | | **TimeD Aerobic-22min** | | **TimeE Aerobic-52min** | | **TimeF Aerobic-292min** | |
| --- | --- | --- | --- | --- | --- | --- | --- | --- | --- | --- | --- | --- |
|  | **Mapping Reads** | **Percentage (%)** | **Mapping Reads** | **Percentage (%)** | **Mapping Reads** | **Percentage (%)** | **Mapping Reads** | **Percentage (%)** | **Mapping Reads** | **Percentage (%)** | **Mapping Reads** | **Percentage (%)** |
| **Metagenomic Assembly** | 53,436,191 | 75.46 | 52,119,320 | 77.55 | 47,295,895 | 76.43 | 50,558,369 | 76.44 | 53,488,638 | 78.24 | 48,598,042 | 77.64 |
|  |  |  |  |  |  |  |  |  |  |  |  |  |
| **Accumulibacter Genomes** |  |  |  |  |  |  |  |  |  |  |  |  |
| Accumulibacter sp. UW-LDO-IC | 34,153,509 | 48.23 | 33,074,188 | 49.21 | 29,322,430 | 47.39 | 30,879,766 | 46.69 | 34,022,590 | 49.77 | 31,266,884 | 49.96 |
| Accumulibacter BA-94 | 339,200 | 0.48 | 301,935 | 0.45 | 255,777 | 0.41 | 346,896 | 0.52 | 317,890 | 0.46 | 224,432 | 0.36 |
| Accumulibacter sp. 66-26 | 269,960 | 0.38 | 293,672 | 0.44 | 245,888 | 0.40 | 333,846 | 0.50 | 237,221 | 0.35 | 178,552 | 0.29 |
| Accumulibacter HKU-1 | 188,368 | 0.27 | 193,623 | 0.29 | 182,887 | 0.30 | 204,697 | 0.31 | 212,465 | 0.31 | 162,371 | 0.26 |
| Accumulibacter sp. SK-01 | 173,780 | 0.25 | 151,800 | 0.23 | 170,589 | 0.28 | 186,542 | 0.28 | 170,390 | 0.25 | 107,689 | 0.17 |
| Accumulibacter UW-1 | 72,429 | 0.10 | 89,236 | 0.13 | 72,698 | 0.12 | 89,727 | 0.14 | 87,761 | 0.13 | 53,462 | 0.09 |
| Accumulibacter UW-2 | 70,297 | 0.10 | 78,092 | 0.12 | 62,826 | 0.10 | 85,618 | 0.13 | 85,442 | 0.12 | 49,247 | 0.08 |
| Accumulibacter sp. BA-91 | 60,110 | 0.08 | 58,687 | 0.09 | 46,351 | 0.07 | 58,856 | 0.09 | 56,933 | 0.08 | 48,747 | 0.08 |
| Accumulibacter isolate UBA2783 | 50,148 | 0.07 | 49,101 | 0.07 | 41,300 | 0.07 | 50,813 | 0.08 | 56,204 | 0.08 | 44,659 | 0.07 |
| Accumulibacter isolate UBA2327 | 49,524 | 0.07 | 44,260 | 0.07 | 37,601 | 0.06 | 48,117 | 0.07 | 49,554 | 0.07 | 41,685 | 0.07 |
| Accumulibacter sp. BA-93 | 38,591 | 0.05 | 35,627 | 0.05 | 31,382 | 0.05 | 40,003 | 0.06 | 35,517 | 0.05 | 34,713 | 0.06 |
| Accumulibacter SK-11 | 38,389 | 0.05 | 30,882 | 0.05 | 30,956 | 0.05 | 35,010 | 0.05 | 34,180 | 0.05 | 34,667 | 0.06 |
| Accumulibacter BA-92 | 32,312 | 0.05 | 28,511 | 0.04 | 26,003 | 0.04 | 28,094 | 0.04 | 27,473 | 0.04 | 28,558 | 0.05 |
| Accumulibacter sp. UBA704 | 16,636 | 0.02 | 17,642 | 0.03 | 15,813 | 0.03 | 19,947 | 0.03 | 13,896 | 0.02 | 12,744 | 0.02 |
| Accumulibacter sp. SK-02 | 13,269 | 0.02 | 15,392 | 0.02 | 10,148 | 0.02 | 14,459 | 0.02 | 13,844 | 0.02 | 9,920 | 0.02 |
| Accumulibacter isolate UBA6585 | 9,822 | 0.01 | 10,203 | 0.02 | 7,879 | 0.01 | 11,236 | 0.02 | 11,501 | 0.02 | 9,355 | 0.01 |
| Accumulibacter isolate HKU-2 | 9,465 | 0.01 | 8,464 | 0.01 | 6,692 | 0.01 | 8,719 | 0.01 | 8,758 | 0.01 | 8,205 | 0.01 |
| Accumulibacter isolate UBA6658 | 7,560 | 0.01 | 6,753 | 0.01 | 5,964 | 0.01 | 6,989 | 0.01 | 6,292 | 0.01 | 5,261 | 0.01 |
| Accumulibacter isolate UBA5574 | 6,600 | 0.01 | 6,686 | 0.01 | 5,256 | 0.01 | 6,188 | 0.01 | 6,113 | 0.01 | 5,054 | 0.01 |
| Accumulibacter isolate UBA2315 | 6,457 | 0.01 | 6,205 | 0.01 | 5,023 | 0.01 | 6,036 | 0.01 | 5,741 | 0.01 | 5,035 | 0.01 |
| Accumulibacter SK-12 | 6,309 | 0.01 | 5,924 | 0.01 | 4,657 | 0.01 | 5,890 | 0.01 | 5,592 | 0.01 | 4,818 | 0.01 |
