# Supplementary material for: Integrated Omic Analyses Provide Evidence that a “Candidatus Accumulibacter phosphatis” Strain Performs Denitrification under Microaerobic Conditions
Source: mSystems. 2019 Jan 15;4(1):e00193-18. doi: 10.1128/mSystems.00193-18 (PMC6446978; doi:10.1128/mSystems.00193-18)
Supplement: TABLE S5 [file mSystems.00193-18-st005.docx]

| **Category** | **Gene** | **66-26** | **UW-1** | **SK-01** | **SK-02** | **BA-91** | **BA-94** | **SK-11** | **SK-12** | **BA-93** | **UW-2** | **BA-92** | **UW-LDO-IC** |
| --- | --- | --- | --- | --- | --- | --- | --- | --- | --- | --- | --- | --- | --- |
| Nitrite/nitrate transport | *narK* |  | CAP2UW1_RS13840 | CAPSK01_00973 CAPSK01_00972 | AW06_03543 AW06_03542 | AW09_04395 AW09_01219 AW09_01220 |  |  | AW08_01696 | AW11_00623 | Ga0078784_16490 | AW10_02293 AW10_04278 AW10_04277 | DVS81_13630 DVS81_13635 |
| Respiratory Nitrate Reduction | *narI* |  |  | CAPSK01_00968 | AW06_03547 | AW09_04883 AW09_04436 |  |  |  |  |  |  | DVS81_13655 |
|  | *narJ* |  |  | CAPSK01_00969 | AW06_03546 | AW09_04435 |  |  |  |  |  |  | DVS81_13650 |
|  | *narH* |  |  | CAPSK01_00970 | AW06_03545 | AW09_03334 AW09_04435 |  |  |  |  |  |  | DVS81_13645 |
|  | *narG* |  |  | CAPSK01_00971 | AW06_03544 | AW09_00845 |  |  |  |  |  |  | DVS81_13640 |
| Periplasmic Nitrate Reduction | *napD* | BGO63_05850 | CAP2UW1_RS19375 |  |  |  | AW12_02777 |  |  | AW11_02156 | Ga0078784_16675 | AW10_00993 |  |
|  | *napA* | BGO63_05855 | CAP2UW1_RS19380 |  |  |  | AW12_02778 |  |  | AW11_02155 | Ga0078784_16676 | AW10_00992 |  |
|  | *napG* | BGO63_05860 | CAP2UW1_RS19385 |  |  |  |  | AW07_00314 | AW08_03811 | AW11_02154 | Ga0078784_16677 | AW10_00991 |  |
|  | *napH* | BGO63_05865 | CAP2UW1_RS19390 |  |  |  |  | AW07_00315 | AW08_03810 | AW11_02153 | Ga0078784_16678 | AW10_00990 |  |
|  | *napB* | BGO63_05870 | CAP2UW1_RS19395 |  |  |  |  |  | AW08_03809 | AW11_02152 | Ga0078784_16679 | AW10_00989 |  |
| Nitrite Reduction | *nirS* | BGO63_10625 BGO63_07995 | CAP2UW1_RS12415 | CAPSK01_00966 | AW06_03549 | AW09_04881 |  | AW07_01971 | AW08_01974 | AW11_03206 AW11_01110 | Ga0078784_11456 Ga0078784_16169 | AW10_00297 AW10_01909 | DVS81_20760 DVS81_12285 DVS81_17305 |
|  | *nirM* | BGO63_10620 | CAP2UW1_RS12410 | CAPSK01_03625 | AW06_03550 | AW09_04517 | AW12_00607 | AW07_01106 | AW08_01973 | AW11_00400 | Ga0078784_11457 | AW10_00298 | DVS81_05230 |
|  | *nirN* | BGO63_15090 | CAP2UW1_RS01495 | CAPSK01_04667 | AW06_00888 AW06_01533 | AW09_05015 AW09_02026 | AW12_01837 | AW07_03428 | AW08_02733 | AW11_00134 | Ga0078784_161768 | AW10_02670 | DVS81_05225 |
|  | *nirJ* | BGO63_15095 | CAP2UW1_RS01500 | CAPSK01_04668 | AW06_01532 AW06_00887 | AW09_02027 | AW12_01838 | AW07_03429 | AW08_02732 | AW11_00135 | Ga0078784_161769 | AW10_02671 | DVS81_12305 |
|  | *nirC* | BGO63_10615 | CAP2UW1_RS12405 | CAPSK01_02537 | AW06_03944 | AW09_01616 |  | AW07_01957 | AW08_01959 | AW11_03210 | Ga0078784_11460 | AW10_00301 | DVS81_16850 |
|  | *nirF* | BGO63_15190 | CAP2UW1_RS16895 | CAPSK01_00714 | AW06_03796 | AW09_02150 | AW12_03398 | AW07_03938 | AW08_02575 | AW11_03018 | Ga0078784_11285 | AW10_00481 | DVS81_16855 |
|  | *nirD/nirL* | BGO63_15185 | CAP2UW1_RS16890 | CAPSK01_00715 | AW06_03795 | AW09_01742 AW09_02149 |  | AW07_03937 | AW08_02576 | AW11_03019 | Ga0078784_11286 | AW10_00481 | DVS81_16860 |
|  | *nirG* | BGO63_15180 | CAP2UW1_RS16885 | CAPSK01_00716 | AW06_03794 | AW09_01741 |  | AW07_03936 | AW08_02577 | AW11_03020 | Ga0078784_11287 | AW10_00480 | DVS81_16865 |
|  | *nirH* | BGO63_15175 | CAP2UW1_RS16880 | CAPSK01_00717 | AW06_03793 | AW09_01740 |  | AW07_03935 | AW08_02578 | AW11_03021 | Ga0078784_11288 | AW10_00479 | DVS81_20925 |
| Nitric Oxide Reduction | *norZ* | BGO63_01415 BGO63_01420 | CAP2UW1_RS11595 |  |  |  |  |  |  | AW11_01319 |  | AW10_01907 | DVS81_16815 |
| Nitrous Oxide Reduction | *nosD* | BGO63_05040 BGO63_15225 | CAP2UW1_RS16930 CAP2UW1_RS19520 |  |  |  | AW12_03390 | AW07_04345 | AW08_03173 | AW11_03011 | Ga0078784_11278 | AW10_01530 AW10_00489 | DVS81_16795 |
|  | *nosZ* | BGO63_01150 | CAP2UW1_RS16950 |  |  |  |  | AW07_04349 | AW08_03048 | AW11_03007 | Ga0078784_11274 | AW10_00493 | DVS81_16830 |
|  | *nosF* | BGO63_15210 | CAP2UW1_RS16915 |  |  |  |  | AW07_03942 | AW08_02570 | AW11_03014 | Ga0078784_11281 | AW10_00484 | DVS81_16835 |
|  | *nosL* | BGO63_15205 | CAP2UW1_RS16910 |  |  |  |  | AW07_03941 | AW08_01972 | AW11_03015 | Ga0078784_11282 | AW10_00485 | DVS81_16840 |
|  | *nosY* | BGO63_15200 | CAP2UW1_RS16905 |  |  |  |  | AW07_03940 | AW08_02572 | AW11_03016 | Ga0078784_11283 | AW10_00484 | DVS81_13630 DVS81_13635 |

| **Category** | **Gene** | **66-26** | **UW-1** | **SK-01** | **SK-02** | **BA-91** | **BA-94** | **SK-11** | **SK-12** | **BA-93** | **UW-IA** | **BA-92** | **UW-LDO-IC** |
| --- | --- | --- | --- | --- | --- | --- | --- | --- | --- | --- | --- | --- | --- |
| Cytochrome c oxidation (*cbb_3_*) | *ccoN* | BGO63_02185 | CAP2UW1_RS12650 | CAPSK01_02001 | AW06_01080 |  | AW12_03440 | AW07_02023 | AW08_01074 | AW11_01636 | Ga0078784_16238 |  | DVS81_08570 |
|  | *ccoO* | BGO63_02190 | CAP2UW1_RS12645 | CAPSK01_02000 | AW06_01081 | AW09_00171 | AW12_03441 | AW07_02022 | AW08_01075 | AW11_01637 | Ga0078784_16237 |  | DVS81_08565 |
|  | *ccoQ* | BGO63_02195 | CAP2UW1_RS12640 | CAPSK01_01999 | AW06_01082 |  | AW12_03442 | AW07_01648 | AW08_01076 | AW11_01638 | Ga0078784_16236 |  | DVS81_08560 |
|  | *ccoP* | BGO63_02200 | CAP2UW1_RS12635 | CAPSK01_01998 | AW06_01083 | AW09_00172 | AW12_03443 | AW07_02020 | AW08_01077 | AW11_01639 | Ga0078784_16235 |  | DVS81_08555 |
|  | *ccoG* | BGO63_02205 | CAP2UW1_RS12630 | CAPSK01_01997 | AW06_01084 | AW09_00173 | AW12_03444 | AW07_02019 | AW08_01078 | AW11_01641 | Ga0078784_16233 |  | DVS81_08545 |
|  | *ccoH* | BGO63_02210 | CAP2UW1_RS12625 | CAPSK01_01996 | AW06_01085 | AW09_00174 | AW12_03445 | AW07_02018 | AW08_01079 | AW11_01642 | Ga0078784_16232 |  | DVS81_08540 |
|  | *ccoI* | BGO63_02175 | CAP2UW1_RS12660 | CAPSK01_02004 | AW06_01078 | AW09_00469 | AW12_01853 | AW07_02026 | AW08_01072 | AW11_01634 | Ga0078784_16240 |  | DVS81_07980 |
|  | *ccoS* | BGO63_02180 | CAP2UW1_RS12655 | CAPSK01_02002 | AW06_01079 |  | AW12_03439 |  | AW08_01073 | AW11_01635 | Ga0078784_16239 |  | DVS81_07975 |
| Cytochrome c oxidation (*ba_3_*) | *cbaA* |  | CAP2UW1_RS08915 | CAPSK01_02286 | AW06_04048 |  | AW12_01750 | AW07_03205 | AW08_03217 | AW11_01056 | Ga0078784_1135 | AW10_02150 | DVS81_11870 |
|  | *cbaB* |  | CAP2UW1_RS08920 | CAPSK01_02287 | AW06_04049 | AW09_03865 | AW12_01749 | AW07_03206 | AW08_03218 | AW11_01057 | Ga0078784_1136 | AW10_02151 | DVS81_11875 |
| Cytochrome c oxidation (*aa_3_*) | *ctaD* | BGO63_08500 BGO63_08555 | CAP2UW1_RS20650 | CAPSK01_02318 | AW06_01187 | AW09_03272 |  |  |  | AW11_00093 | Ga0078784_161727 | AW10_02621 | DVS81_11220 |
|  | *ctaC* | BGO63_08495 BGO63_08550 | CAP2UW1_RS20655 | CAPSK01_02319 | AW06_01186 | AW09_03273 AW09_03276 |  | AW07_03129 | AW08_03918 | AW11_00092 | Ga0078784_161724 | AW10_02617 AW10_02620 | DVS81_11215 |
|  | *ctaE* | BGO63_08505 BGO63_08565 | CAP2UW1_RS20640 | CAPSK01_02314 | AW06_01191 | AW09_03269 | AW12_00507 | AW07_01646 | AW08_03349 | AW11_00097 | Ga0078784_161731 | AW10_02625 | DVS81_11235 |
